# Synergistic effects of bioactive glass and sodium alginate on the surface properties and therapeutic release of ciprofloxacin from apatite cements

**DOI:** 10.1016/j.ijpx.2025.100401

**Published:** 2025-09-19

**Authors:** Hanaa Mabroum, Hamid Ait Said, Hamza Elbaza, Yousra Hamdan, Said Zayane, Rachid Hakkou, Sanae Ben Mkaddem, Rachid El Fatimy, Hicham Ben Youcef, Hassane Oudadesse, Hassan Noukrati, Allal Barroug

**Affiliations:** a Faculty of Medical Sciences, UM6P Hospitals, Mohammed VI Polytechnic University, Benguerir 43150, Morocco; b High Throughput Multidisciplinary Research Laboratory (HTMR), Mohammed VI Polytechnic University (UM6P), Benguerir, Morocco; c Laboratory of Innovative Materials, Energy and Sustainable Development (IMED-Lab), Faculty of Science and Technology Gueliz, Cadi Ayyad University (UCA), Marrakech, Morocco; d Geology and Sustainable Mining Institute (GSMI), Mohammed VI Polytechnic University (UM6P), Benguerir, Morocco; e Univ Rennes, CNRS, ISCR-UMR 6226, F-35000 Rennes, France; f Cadi Ayyad University, Faculty of Sciences Semlalia, 2390, 40000 Marrakech, Morocco

**Keywords:** Bioactive glass, Alginate, Apatite cement, Adsorption, Release kinetics, Antibacterial activity, Cytotoxicity

## Abstract

This work aims to investigate the effect of the incorporation of additives, including 46S6 bioactive glass (BG) and sodium alginate polymer (Alg), on the adsorption behavior, drug release kinetics, *in vitro* degradability, antibacterial activity, cytotoxicity, and inflammatory response of ciprofloxacin (Cip)-loaded reference cement (RC). Microstructural analysis revealed that the addition of BG and Alg to the reference cement composition (DCPD-CaCO_3_) significantly affected its specific surface area, porosity, surface charge, and the pH of the cement pastes, as well as the solubility of ciprofloxacin within the cement matrix. The adsorption and release behaviors of ciprofloxacin were examined in relation to these modified cement properties and the physicochemical characteristics of ciprofloxacin. The results revealed that the adsorption process was mainly governed by a Freundlich-type isotherm, which is characterized by a low affinity between the Cip molecules and the carrier surface. Moreover, the results of the antibiotic release showed that antibiotic release is influenced mainly by the pH and solubility of Cip. Depending on the composition of the cement, the release follows mechanisms driven by Fick's law of diffusion alone or in combination with other mechanisms. The *in vitro* biodegradation test of the prepared cements in phosphate buffer solution attested that adding BG and alginate improved the degradability of the reference cement. Moreover, the formulated cements exhibited good antibacterial activity against *Staphylococcus aureus* and *Escherichia coli*. Finally, the *in vitro* investigation revealed the non-cytotoxicity and non-inflammatory effects of the ciprofloxacin-loaded cements towards hPBMCs cells, confirming their biocompatibility.

## Graphical Abstract

**Figure:**
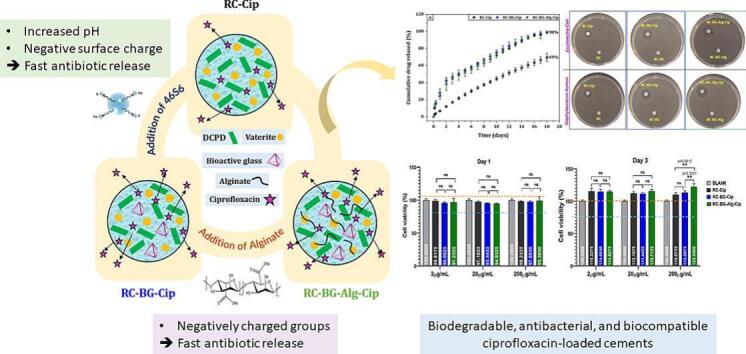

## 1 Introduction

Every year, millions of patients worldwide require bone grafting surgery due to bone diseases, such as bone cancer, osteoporosis, and accidents (Boskey and Imbert, 2017). During bone grafting surgery, the risk of postoperative infection is difficult to prevent and often requires additional antibiotic treatment administered either orally or intravenously (Gomes et al., 2013; Nandi et al., 2016). However, both approaches have several drawbacks, including poor bone penetration and the presence of side effects(Thabit et al., 2019). To enhance the prognosis of these patients, local delivery of antibiotics has emerged (Kar et al., 2014; Otsuka and Otsuka, 2005; Wei et al., 2021). Several approaches have focused on developing multifunctional therapeutic materials that can simultaneously promote bone regeneration and ensure localized delivery, sustained antibiotics release for treating bone infections such as osteomyelitis disease (Antipov et al., 2025; Lin et al., 2024; Mistry et al., 2025).

Among these materials, self-setting calcium phosphates (CPCs) have been used extensively as biomaterials to fill bone defects due to their biological properties and ability to set at physiological temperature (Dima et al., 2024; Yousefi, 2019). They are frequently designed in an injectable form to facilitate their use in minimally invasive procedures (Zhou et al., 2019). In addition, CPC-based delivery systems can locally administer drugs with controlled release profiles and high bioavailability (Lukina et al., 2023; Nayak et al., 2024; van Houdt et al., 2018). Thus, the active species are released locally for a prolonged period in the implantation site, which helps prevent and treat infections that may occur during and after surgical procedures.

Several factors could influence the drug release from calcium phosphate cement, including matrix composition, additives, drug solubility and concentrations, drug loading methods, and release media (Fosca et al., 2022). Introducing additives such as polymers (Dusim et al., 2025; Kamitakahara et al., 2025) and bioactive glasses (BGs) (Abd El-Hamid et al., 2024; Demir et al., 2024; Onofrio et al., 2024), which could imply modifications in the microstructure of the reference matrix, is one of the explored approaches for achieving controlled and sustained drug release. BGs are also widely explored as a drug delivery carrier for biomolecules like antibiotics (Almasri and Dahman, 2025; El-Kady et al., 2021), anti-inflammatory drugs, growth factors, proteins, enzymes, and genes (Demir-Oğuz et al., 2023; Fiume et al., 2018). Furthermore, biopolymers, specifically polysaccharides, have proven their efficiency as drug-delivery systems (Barclay et al., 2019; Gopinath et al., 2018). Sodium alginate is one of the most exploited biopolymers for designing drug delivery systems (Hariyadi and Islam, 2020). The reported literature shows the wide application of alginates for encapsulating/loading and delivering various types of biomolecules to treat different diseases (Chaturvedi et al., 2019).

Beyond material formulation, understanding the interaction mechanisms between the matrix and the drug could be considered a key factor in the release mechanism. The adsorption phenomenon has been considered an appealing approach for better understanding the calcium phosphate matrix-drug interactions, thereby controlling the load and elution of drug molecules from the carrier matrix (Rey et al., 2014). Although the mechanisms governing drug release from CPCs have been extensively studied, little attention has been paid to the mechanisms of CPC-drug interactions, more specifically, the adsorption of drugs at CPC interfaces.

The current research aims to design multifunctional antibacterial composite cements for use as bone substitutes and carriers of antibiotics for the local treatment and prevention of postoperative-related infections. The investigated composite cements were prepared by combining dicalcium phosphate dihydrate (DCPD), calcium carbonate, silica-based bioactive glass (46S6), sodium alginate polymer, and ciprofloxacin (Cip) antibiotic. The effect of these components on the adsorption capacity of a reference cement (DCPD-CaCO_3_) was systematically investigated to elucidate the key interactions governing ciprofloxacin binding with the cement's matrix. Furthermore, the release kinetics of ciprofloxacin were studied in relation to the matrix composition and the surface-interfacial properties. The influence of these components on the degradability, as well as the antibacterial efficacy, cytotoxicity, and inflammatory response of the prepared cements, was also investigated. To the best of the author's knowledge, this study is the first to investigate the synergistic effects of bioactive glass and alginate on the functional surface-interface properties and the release of ciprofloxacin from apatite cements.

## 2 Materials and methods

### 2.1 Cements formulation

The composite cements were formulated according to the protocol described in our previous publication (Mabroum et al., 2021). Briefly, the formulation process involved mixing the powder phase composed of vaterite (CaCO_3_), brushite (DCPD, CaHPO_4_·2H_2_O), and 46S6 bioactive glass (BG), with distilled water or alginate gel at an L/P ratio of 0.7. The BG used in this study had the following composition: 6 wt% P_2_O_5_, 24 wt% Na_2_O, 24 wt% CaO, and 46 wt% SiO_2_, with a particle size between 100 and 200 μm (particle size distribution shown in Supplementary Information, Fig. S1). The Cip-loaded cements were prepared by incorporating the powder drug (3 % by weight) into the reactive powders. Then, the obtained mixture was combined with distilled water or alginate gel.

The prepared pastes were placed in a polyethylene tube immediately after preparation in 100 % relative humidity to mature and set for 48 h at 37 °C, followed by 5 days of drying at 37 °C. The composition of the formulated systems is shown in Table 1. The composition of the solid phase is expressed as a percentage (%) by weight. The selected formulation (25 wt% BG, 5 wt% Alg, and.

**Table 1:** Composition of the prepared cements.

Cements	Solid phase (wt%)				Liquid phase	L/P
	CaCO_3_	DCPD	BG	Cip		
RC	50	50	0	0	Distilled water	0.7
RC-BG	37.5	37.5	25			
RC-BG-Alg	37.5	37.5	25		Alg gel (5 wt%) in distilled water	
RC-Cip	48.5	48.5	0	3	Distilled water	
RC-BG-Cip	36	36	25			
RC-BG-Alg-Cip	36	36	25		5 wt% Alg in distilled water	

L/*P* = 0.7) corresponds to the optimized composition identified in our previous studies (Mabroum et al., 2021).

### 2.2 Characterization of cements without and with ciprofloxacin

The cements with and without ciprofloxacin were characterized using various and complementary techniques. The diffractometer Rikagu D/Max-IIIB (CuKα source) was used to assess the phase structure. Data were recorded from 10° to 70° (2θ). Origin software was used to calculate the peak area ratio of apatite to vaterite (A_A_/A_V_). The “A_A_” and “A_V_” terms denote the XRD peak areas of precipitated apatite and residual vaterite, respectively. Since the most intense peaks overlapped, peak areas were calculated from the more distinct peaks at 25.1° (*V*) and 26.1° (A). A Nicolet 5700 spectrometer was used for FTIR analysis at room temperature, and the generated spectra were collected in the wavenumber range of 4000–400 cm-1 with a resolution of 4 cm^−1^. The TESCAN VEGA3 scanning electron microscope was used to investigate the morphology of cements coated with a thin, conductive carbon layer.

The 3 Flex adsorption analyzer from Micromeritics (Norcross, GA, USA) was used to determine the Nitrogen (N_2_) adsorption/desorption isotherms. For these measurements, the set cement samples were first ground into powders and sieved (<50 μm). Powder masses ranging from 30 to 80 mg were used for the analyses. Before the measurement, cements samples were outgassed overnight at room temperature in a Smart VacPrep degas system. The obtained isotherms from the Brunauer-Emmett-Teller (BET) equation allowed the determination of the specific surface area. Five experimental points from the linear range of the BET plot were used. Furthermore, the Barrett-Joyner-Halenda (BJH) analysis method was used to evaluate the mesopore size distribution.

The Micromeritics AutoPore IV 9500 V1.09 mercury porosimeter was used to examine Mercury Intrusion Porosity (MIP). For these measurements, set cement blocks were used, with sample masses of approximately 0.1 g.

Evaluation of the zeta potential of the prepared cements was performed using a Zeta-sizer (Nano ZS90, Malvern Instruments Ltd). The set cements were ground manually with an agate mortar and pestle and sieved through a 50 μm mesh to obtain powders with particle size <50 μm. Suspensions were prepared by dispersing cement powders (0.01 wt% with a particle size <50 μm) in 1 mM potassium chloride (KCl) solution with different initial pH values regulated. The acidic pH was regulated (pH ≈ 4) by adding hydrochloric acid solution (HCl) with a concentration of 0.1 M, and the basic (pH ≈ 8) and neutral pH (pH ≈ 7) were obtained using sodium hydroxide solution (NaOH) at a concentration of 0.1 M. After sonication, the suspensions were allowed to settle overnight, and the equilibrium pH was determined. The KCl solution (1 mM) was used as the background electrolyte. The values obtained in this study were calculated as the average of three repeated tests. It should be noted that under acidic conditions (pH ≈ 4), partial dissolution of BG may occur; therefore, the reported zeta potential values represent the equilibrated surface charge of the cement particles under the tested conditions rather than the pristine surface of the as-prepared material.

Although the study focuses on analyzing interfacial properties, complementary measurements of setting time, injectability, compressive strength, and bioactivity were also performed. These results are presented in Supplementary Information (Figs. S3 and S4).

### 2.3 Adsorption study

The ciprofloxacin (Cip) adsorption assays by RC, RC-BG, and RC-BG-Alg powders were conducted in a KCl solution at room temperature. The Ciprofloxacin powder was dissolved in potassium chloride (KCl, 1 mM, pH 7) to obtain the drug solution. The kinetic study was performed by dispersing 25 mg of the adsorbent in a ciprofloxacin solution (5 mL) at a concentration of 1.5 mg/L. The suspensions were sonicated for 10 min in continuous mode at a frequency of 50 Hz, then incubated at room temperature for various durations ranging from 15 to 1440 min. The adsorption isotherm was built at a fixed contact time by plotting the amount of Cip adsorbed by the solid as a function of its equilibrium concentration; the Cip concentrations investigated ranged from 0 to 30 mg/L. The suspensions were then centrifuged after 6 h of incubation, the Cip concentration in the supernatant was determined, and the antibiotic-free solutions were used as a blank control.

For simple comparison, adsorption tests were conducted under the same conditions using the parent reagents (bioactive glass and vaterite) of the formulated cements. All sorption tests were conducted three times and were reproducible within a range of 6 %. The following equations (Eq. (1) and Eq. (2)) were used to calculate the Cip adsorbed amount (Q_Ads_) and the adsorption efficiency (Ads (%)):

(1) $$\;Q_{\mathrm{Ads}}=V*\frac{\left(C_{0}-C_{\mathrm{eq}}\right)}{m}$$

(2) $$\mathrm{Ads}\;\left(\%\right)=\frac{\left(C_{0}-C_{\mathrm{eq}}\right)}{C_{0}}*100$$

where the terms Q_Ads_ (mg/g) and Ads (%) are the antibiotic amount adsorbed and the adsorption efficiency, respectively. The terms C_o_ and C_eq_ (mg.L^−1^) refer to the starting and equilibrium concentrations of the adsorbate, respectively. *V* (L) states the volume of the adsorption medium, while m (g) represents the amount of the solid.

The Cip concentration was quantified *via* UV–Visible spectrophotometry at λ_max_ of 271 nm. Calibration curves (R^2^ ∼ 0.99) were constructed over the concentration range of 0 to 8 mg/L (Fig. S2). The extinction coefficient value was 0.1203 L.mg^−1^.cm^−1^.

### 2.4 Antibiotic release study

Using Dissolutest equipment (Pharmatest®), the antibiotic release assays were conducted according to the protocol described in our previous work (Mabroum et al., 2023).

Ciprofloxacin-loaded cements were prepared in cylindrical shapes (10 mm diameter × 10 mm height) and immersed in PBS medium (pH 7.4) under stirring conditions. A volume of 5 mL of solution was sampled daily. The released antibiotic quantities were analyzed by UV spectrophotometry at 271 nm. The cumulative rate of released ciprofloxacin (Q) was determined using the following equation:

(3) $$Q=\frac{w_{t}}{w_{0}}*100$$

Where w_0_ and w_t_ are, respectively, the initial quantity of Cip added in the cements and the amount released (mg) from the cements at a time t.

The mathematical models of zero-order (Eq. (4)), Korsmeyer-Peppas (Eq. (5)), and Higuchi (Eq. (6)) were employed to determine the mechanisms governing the release kinetics of ciprofloxacin from the designed composite cements.

**The zero-order kinetic** is applied when drug dissolution is solely a matter of time and occurs regardless of the drug concentration at any particular time point (Askarizadeh et al., 2023; Pastorino et al., 2015). The zero-order equation can be mathematically represented as follows:

(4) $$Q=K_{0}t$$

Q is the fraction of the dissolved drug at time t, and K_0_ is the constant release rate that does not change during the dissolution process.

**The Korsmeyer-Peppas** equation is a semi-empirical model used generally to characterize drug elution from monolithic or polymeric systems. Korsmeyer-Peppas is represented by Eq. (5).

(5) $$Q=\frac{M_{t}}{M_{0}}=k_{\mathrm{KP}}t^{n}$$

where Q (or M_t_/M_o_) is the released fraction of the drug, “k_KP_” is the kinetic constant, “n” is the diffusion coefficient, and “t” is the release time (min). The parameter “n” indicates the drug elution process, which depends on the dimensions of the delivery material. For the cements that are cylindrical, the Fickian diffusion is the process governing the release when *n* ≤ 0.45; the coexistence of erosion and diffusion phenomena occurs when 0.45 < *n* < 1 (Korsmqer et al., 1983; Paarakh et al., 2019).

The Higuchi model (Eq. (6)) describes the relationship between the cumulative rate of drugs released and the square root of time (Siepmann and Peppas, 2011).

(6) $$Q=\frac{M_{t}}{M_{0}}=K_{H}t^{0.5}$$

Higuchi's diffusion constant is represented by the parameter “K_H_” in the equation (Eq. (6)).

### 2.5 Characterization of composite cements after the release test

Following the release test, the cements (RC, RC-BG, and RC-BG-Alg) were characterized by FTIR and XRD to assess the evolution of their microstructure and composition.

The *in vitro* biodegradability of the composite cements was investigated by monitoring the concentrations of calcium (Ca) and silica (Si) ions released from the prepared materials during the ciprofloxacin release test. The elements were analyzed using inductively coupled plasma mass spectrometry (ICP-AES, PerkinElmer Optima 7000 DV). Furthermore, the weight loss percentage was determined by weighing the cement specimens before and after the release test, after they were washed and dried. The average value was presented after testing three samples of each cement. The weight loss rate was calculated using the following formula:

(7) $$\text{Weight loss rate}=\frac{W_{0\;}-W_{t}}{W_{0}}*100\%$$

W_0_ and Wt are the weights before and after the release test, respectively.

Compressive strength tests were performed on cement samples before and after immersion in the release medium (PBS solution, pH 7.4). Cylindrical monoliths (16 mm × 8 mm) were prepared, matured for 2 days at 37 °C in a humid environment, and subsequently dried for 5 days at 37 °C. The dried monoliths were then immersed in PBS solution for 18 days. The mechanical tests were conducted using a universal testing machine at a crosshead speed of 1 mm/min, and the results are expressed as the mean ± standard deviation (*n* = 3).

### 2.6 Antibacterial activity

The antibacterial efficiency of ciprofloxacin-loaded cements was *in vitro* assessed by the disk diffusion test on *Escherichia coli* and *Staphylococcus aureus* germs. The detailed protocol was described in our previous paper (Mabroum et al., 2023). Briefly, cylindrical composite cements were immersed in sterilized ultrapure water. The supernatants were then placed on absorbent filter paper disks and allowed to soak for a short time. The bacteria were grown in a tryptone soy broth medium (TSB) with agitation and then diluted in TSB to form the bacterial culture. After that, the bacteria were incubated at room temperature in sterile Petri dishes until solidified. The bacteria-infected Petri dishes were then covered with filter paper discs containing the supernatant. Samples without ciprofloxacin were used as controls. Photographs were taken after 24 h of incubation, and then the bacterial inhibition diameter was calculated according to the following formula (Eq. (8)):

(8) $$\text{Inhibition zone diameter}\;\left(\mathrm{mm}\right)=\frac{\left({Ø}_{\mathrm{iz}}-{Ø}_{d}\right)}{2}$$

Ø_iz_ (mm) is the inhibitory zone diameter, and Ø_d_ (mm) is the diameter of the filter paper disc.

### 2.7 *In vitro* cytotoxicity

#### 2.7.1 Separation and cell viability of hPBMCs

10 mL and 35 mL of peripheral blood were collected from two healthy, voluntary consent donors (a 35-year-old and a 28-year-old) following ethical committee approval. The isolation of hPBMCs was performed following the protocol published in our previous work (Noukrati et al., 2024).

The MTT assay was conducted to assess hPBMCs' viability in response to various cement compositions (RC-Cip, RC-BG-Cip, and RC-BG-Alg-Cip). The cement powders with different concentrations (200 μg/mL, 20 μg/mL, and 2 μg/mL) were sterilized for 2 h using UV treatment. The extracted hPBMCs were placed in three separate 96-well plates at a density of 10^4^ cells per well, using 100 μL of RPMI medium supplemented with 10 % FBS and 1 % antibiotics, and then incubated overnight. Next, sterilized cement powders (RC-Cip, RC-BG-Cip, and RC-BG-Alg-Cip) were added to the medium and incubated at 37 °C overnight. For each concentration, quadruplicate samples of 100 μL were introduced into the wells and cultured (at 37 °C with 5 % CO_2_) in a humidified atmosphere for varying durations. After 1 and 3 days of culture, 10 μL of MTT solution (VWR, USA) was then added to each well and incubated for 3 h at 37 °C. Formazan crystals were solubilized with 100 μL of dimethyl sulfoxide (DMSO) and incubated for 3 h. FLUOstar Omega Microplate Reader (BMG Labtech) was used to determine the absorbance at 570 nm. A baseline value of 100 % viability was assigned to the untreated cells, which served as a negative control group, and cell viability was expressed as a percentage of these cells. The results obtained in the presence of cements were normalized to the absorbance of the blank and expressed as a rate according to the equation:

(9) $$\mathrm{Cell viability}\;\left(\%\right)=\frac{\mathrm{number of treated cells}}{\mathrm{number of blank cells}}x\;100$$

#### 2.7.2 Enzyme-linked immunosorbent assay for IL-8 measurement

In a 48-well plate with full medium, 1.6 million hPBMCs were seeded per well. The plates were then incubated overnight at 37 °C in a humid environment with 5 % CO_2_. Based on the cytotoxicity results, a cement powder concentration of 200 μg/mL was selected for this experiment, using different cement formulations (RC-Cip, RC-BG-Cip, and RC-BG-Alg-Cip). Lipopolysaccharide (LPS) was used as a positive control to stimulate the hPBMCs, while untreated wells containing only cells and medium served as the negative control. ELISA (Enzyme-linked immunosorbent assay) kits (R&D Systems) were used to quantify the amounts of interleukin-8 (IL-8) in the supernatants of stimulated cells, following treatment, with absorbance measured at 450 nm.

#### 2.7.3 Statistical analysis

A statistical analysis was performed on the data relating to cytotoxicity (cell viability) and inflammatory response (Enzyme-linked immunosorbent assay for IL-8 measurement).

The reported values are presented as the mean ± standard deviation (SD). Statistical analysis was performed using GraphPad Prism 8.0, followed by a one-way ANOVA with Dunnett's multiple comparison tests to assess the effects of the treatment groups compared to the control group. A significance threshold was set at *p* < 0.05.

#### 2.7.4 Ethical approval

Following the guidelines set forth by the Moroccan Ministries of Health and Higher Education, the isolation and use of human peripheral blood mononuclear cells (hPBMCs) used in this study have been approved by the Ethics Committee of the Mohammed VI University Hospital and by the Faculty of Medicine and Pharmacy at Cadi Ayyad University (Ethical approval code number: 36/2023). This approval was granted within the framework of the project entitled “Valorization of Phosphorus for Regenerative Medicine: Application to Cartilage Repair in Arthritic Pathologies.”

## 3 Results & discussion

### 3.1 Characterization of cements without and with ciprofloxacin

#### 3.1.1 XRD and FTIR analysis

**Fig. 1:**
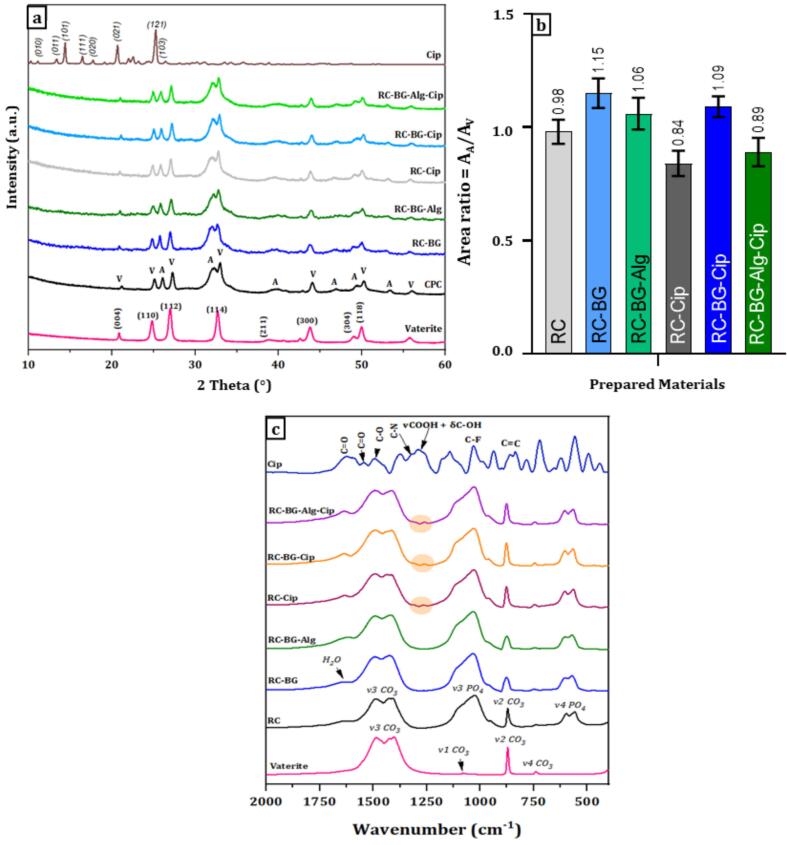
XRD patterns (a), FTIR spectra (b) of ciprofloxacin, vaterite, reference cement (RC) composite cements without ciprofloxacin (RC-BG, RC-BG-Alg) compared to ciprofloxacin-loaded cements (RC-Cip, RC-BG-Cip, RC-BG-Alg-Cip), (c) area ratio (A_A_**/**A_V_) of cements without ciprofloxacin (RC, RC-BG, RC-BG-Alg) compared to ciprofloxacin-loaded cements (RC-Cip, RC-BG-Cip, RC-BG-Alg-Cip).

Fig. 1 depicts the XRD diagrams (a), the area ratio (b), and FTIR spectra (c) of the reference cement (RC) compared to vaterite precursor, the composite cements without ciprofloxacin (RC-BG, RC-BG-Alg), and ciprofloxacin-loaded cements (RC-Cip, RC-BG-Cip, RC-BG-Alg-Cip).

The X-ray diffractogram of reference cement (RC) showed peaks typical of newly formed apatite and vaterite phases (Fig. 1.a). The most intense apatite phase peaks were detected at 26.1° and 32.2° while vaterite peaks were identified at 21.16°, 25.1°, 27.3°, and 33.0° (JCPDS datafile # 01–072-0506). Brushite peaks, on the other hand, were not visible in the XRD pattern, indicating that the DCPD phase reacted completely during the setting reaction as described in the chemical equation (Eq. (10)) (Mabroum et al., 2022; Mabroum et al., 2021).

(10) $${\text{CaHPO}}_{4},{\text{2H}}_{2}O+{\text{CaCO}}_{3}➜{\mathrm{Ca}}_{10-x}\left(\mathrm{PO}4\right)6-x{\left(\text{CO3}\right)}_{x}\left(\mathrm{OH}\right)2-x+{\text{CaCO}}_{3}\;\left(\text{excess}\right)$$

The XRD patterns of the composite cements RC-BG and RC-BG-Alg were similar to that of the RC cement; thus, the primary peaks of residual vaterite and precipitated apatite were identified, and no further phases were found after the addition of bioactive glass and alginate polymer.

Adding ciprofloxacin to RC, RC-BG, and RC-BG-Alg cements did not affect their structure. Indeed, XRD diagrams of ciprofloxacin-loaded cements (RC-Cip, RC-BG-Cip, RC-BG-Alg-Cip) were similar, indicating the presence of the same phases, apatite, and vaterite, with no detected peak of brushite and ciprofloxacin antibiotic.

To assess apatite formation during the setting reaction of the prepared cements, the A_A_/A_V_ area ratio of the XRD peaks corresponding to apatite (25.8°) and vaterite (24.8°) was determined using Origin software. Fig. 1.b depicts the A_A_/A_V_ area ratio for cements without ciprofloxacin (RC-BG, RC-BG-Alg) compared to ciprofloxacin-loaded cements (RC-Cip, RC-BG-Cip, RC-BG-Alg-Cip). The results revealed that the RC cement exhibited an area ratio of 0.98, which increased to 1.15 for RC-BG cement and slightly decreased to 1.06 for the cement containing bioactive glass and sodium alginate (RC-BG-Alg). These values indicated that the chemical composition of the RC after setting and hardening exhibited the highest amount of vaterite or the lowest amount of apatite, followed by RC-BG-Alg. The RC-BG cement had the lowest amount of vaterite or the highest amount of apatite.

The incorporation of 3 wt% of ciprofloxacin antibiotic into the cements resulted in a decrease in the area ratio (A_A_/A_V_), attesting that the ciprofloxacin altered the apatite formation. Indeed, the area ratio decreased from 0.98 for RC to 0.84 for RC-Cip, and from 1.15 for RC-BG to 1.09 for RC-BG-Cip. This effect was also noticed for the composite cement, attesting that the area ratio increased from 1.06 for RC-BG-Alg to 0.89 for RC-BG-Alg-Cip. This trend may be attributed to a competitive reaction between the calcium ions (positively charged) present in the cements and ciprofloxacin molecules, especially through the carboxyl group R-COOH, resulting in “chelation” and subsequent consumption of the calcium ions.

The FTIR spectra (Fig. 1.c) of composite cements without ciprofloxacin (RC-BG and RC-BG-Alg) are similar, showing the presence of bands typical of carbonated apatite as well as the presence of carbonate groups of vaterite (Al Omari et al., 2016; Mabroum et al., 2021). Indeed, phosphate bands of the apatite phase in the range between 470 and 620 cm^−1^, which are attributed to PO_4_^3−^ and HPO_4_^2−^ ions (Rey et al., 2017). The apatite bands corresponding to ʋ_3_PO_4_ were observed in the wavenumber range 1000–1105 cm^−1^. The bands observed at 745, 875, and 1440–1490 cm^−1^ were assigned to the carbonate groups of the residual vaterite as well as to carbonates in the newly formed carbonated apatite phase (Combes et al., 2006). In addition, the FTIR spectrum of RC-BG revealed the appearance of the Si-O-Si band typical of the presence of bioactive glass incorporated into the reference cement. However, no bands of alginate were detected (Mabroum et al., 2022; Mabroum et al., 2021). Furthermore, the FTIR spectra of ciprofloxacin-loaded cements (RC-Cip, RC-BG-Cip, RC-BG-Alg-Cip) showed the appearance of new bands characterizing the ciprofloxacin molecule in the region 779–825 cm^−1^, 1261 cm^−1^, 1305 cm^−1^, attributed to the C=C, vCOOH, δC-OH, and C-N vibrations, respectively. The band observed at 1618 cm^−1^ could be attributed to both residual water bonding vibrations and the C=O group of the alginate.

#### 3.1.2 Structural, morphological, and textural characterization

**Fig. 2:**
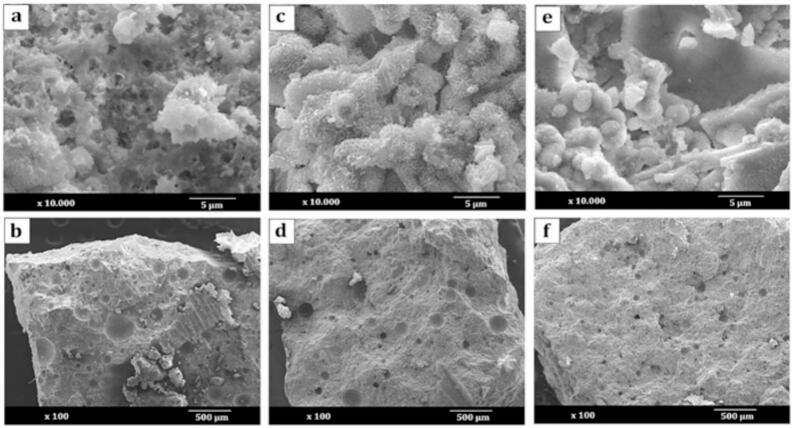
SEM micrographs at 5 and 500 μm of RC-Cip (a and b), RC-BG-Cip (c and d), and RC-BG-Alg-Cip (e-f) cements.

The hardened cements' morphology was examined by SEM at a 100 μm scale (Fig. 2.a, b, c, d, e, and f). The SEM images of Cip-loaded reference cement (RC-Cip) showed the presence of large pores (Fig. 2.b), and the incorporation of BG (RC-BG-Cip) resulted in fewer pores with a slight decrease in the pore diameter (Fig. 2.d). The SEM micrograph of RC-BG-Alg-Cip cement (Fig. 2.f) showed the densest structure with the lowest pore diameter.

Furthermore, the images captured at magnifications of 10.000 (Fig. 2.b, d, and f) revealed the presence of microporosity in the RC-Cip, and when BG and Alg were added, the structure became denser. Moreover, the SEM image of RC-BG-Cip cement showed fine needle crystals typical of the apatite phase and particles in the form of blocks corresponding to the bioactive glass for the RC-BG-Alg-Cip cement.

**Fig. 3:**
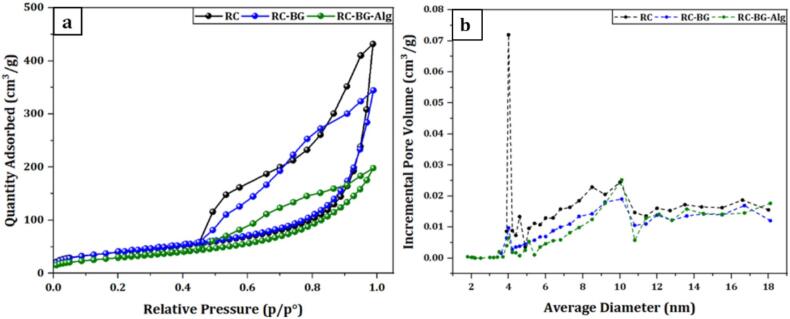
N2 adsorption-desorption isotherms (a) and pore size distribution (b) of prepared materials.

To evaluate the textural parameters of formulated cements, the N_2_ adsorption−desorption method was used. Fig. 3.a depicts the N_2_ adsorption-desorption isotherms of the cements' powders (≤ 50 μm) formulated without ciprofloxacin. The curves of both samples could be characterized as IV isotherm type with an H3 hysteresis loop, as classified by IUPAC, suggesting their mesoporous structures. Furthermore, the incorporation of BG and Alg into the RC cement affected the isotherms' shape. The N_2_ adsorption-desorption isotherms became less large, revealing the reduction of the specific surface area from 149 m^2^.g^−1^ for RC to 145 m^2^.g^−1^ for RC-BG and 110 m^2^.g^−1^ for RC-BG-Alg cement. This decrease in the SSA was associated with a decrease in the average pore size, which decreased from 16.6 nm for RC to 13.3 and 10.6 nm for RC-BG and RC-BG-Alg cements, respectively (Table 2). The same tendency has been reported by Oznur et al., who investigated the effect of the particle size on the SSA of an α-TCP cement, and attested that increasing the particle size of mesoporous bioactive glass (58S, prepared by the sol-gel process) led to a decrease in the specific surface area (Demir et al., 2024).

**Table 2:** SSA and porosity values of prepared cements (RC, RC-BG, and RC-BG-Alg), vaterite, and bioactive glass (BG).

Prepared Materials	SSA (m^2^.g^−1^)*	Average pore diameter (nm)*	Porosity (MIP%)**	Pore volume (cm^3^.g^−1^)**
BG	0.29	12.1	–	–
Vaterite	21	44.2	–	–
RC	149	16.6	51.6	0.60
RC-BG	145	13.3	48.0	0.49
RC-BG-Alg	110	10.6	43.5	0.29

BET* and MIP**.

Fig. 3.b plots pore volume distribution *versus* mean pore diameter for RC, RC-BG, and RC-BG-Alg cements. The RC sample exhibited a large peak at ∼4 nm, indicating a high number of small mesopores. However, this peak was considerably reduced in the RC-BG and RC-BG-Alg samples, demonstrating partial pore filling or blocking after incorporating BG and BG-Alg. All cements showed additional peaks between ∼6–18 nm, indicating a broader mesoporous distribution. Minor shifts and differences in intensity indicate changes in textural properties induced by incorporating bioactive glass and alginate as additives.

The measurements of the porosity and pore volume determined by Mercury Intrusion Porosity (MIP) on the cements' blocks are depicted in Table 2. The obtained results showed that RC cement exhibited a more porous structure with a porosity value of 51.6 % and a pore volume of 0.60 cm^3^.g^−1^. The porosity was affected by the incorporation of bioactive glass, resulting in a decrease in porosity from 51.6 % for RC to 48 % for the RC-BG composite cement. The same trend was also observed for the pore volume, which decreased from 0.60 cm^3^.g^-1^ for RC to 0.49 cm^3^.g^-1^ for RC-BG. Moreover, the addition of sodium alginate polymer to the cement matrix resulted in the lowest porosity, with a value of 43.5 %, and a total pore volume of 0.29 cm^3^.g^-1^, revealing that the RC-BG-Alg composite cement had the densest structure. The effect of BG on porosity can be attributed to the precipitation of a higher amount of apatite, which fills the pores and voids between particles. The use of alginate as a gel acted as a binder, filling the pores and decreasing porosity. The ionic exchange between sodium and calcium ions led to the formation of an insoluble complex, known as calcium alginate, which affects porosity by forming a cross-linking network, resulting in the densest structure.

#### 3.1.3 Potential zeta measurements

**Fig. 4:**
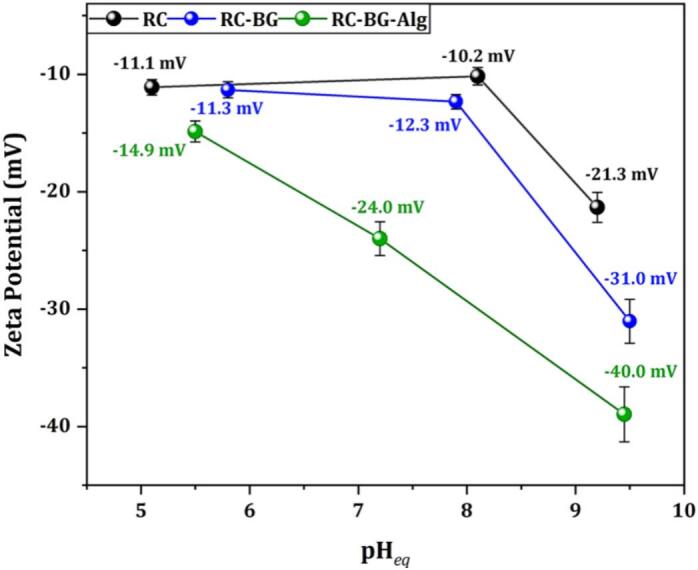
Zeta potential values of RC, RC-BG, and RC-BG-Alg. Results are presented as mean ± SD (*n* = 3). Relative standard deviation (RSD%) values ranged as follows: RC (2.77 % - 8.09 %), RC-BG (1.45 % -12.36 %), and RC-BG-Alg (1.83 % - 4.98 %).

The prepared cements were subjected to zeta potential measurements (Fig. 4) to assess the effect of adding BG and Alg on the surface charge of the reference cement.

The results showed that all the cements developed had a negatively charged surface, despite the pH of the suspension medium. As the solution's pH increased, the negative charge increased. For all examined pH levels, the highest negative surface was obtained for RC-BG-Alg (from −14.9 to −40.0 mV), followed by RC-BG (−11.3 to −31.0 mV) and RC (−11.5 to −21.3 mV). The negative charge of the reference cement surface can be attributed to the presence of carbonate ions (${\mathrm{CO}}_{3}^{2-}$), phosphate ions (${\mathrm{HPO}}_{4}^{2-}$ and ${\mathrm{PO}}_{4}^{3-}$), and hydroxide ions (OH^−^) of the carbonated apatite exposed on their surface. The state of the phosphate groups was pH-dependent and could change from ${\mathrm{HPO}}_{4}^{2-}$ to $H_{2}{\mathrm{PO}}_{4}^{-}$, and the carbonate groups changed from ${\mathrm{CO}}_{3}^{2-}$ to $H{\mathrm{CO}}_{3}^{-}$, as the pH decreased, thereby modifying the overall charge of the cement surface. Moreover, the addition of BG resulted in a higher negative surface charge due to the presence of silanols (Si-OH), which deprotonate at higher pH and generate a high negative surface charge (negatively charged silicate species) (Doostmohammadi et al., 2011; Lu et al., 2000).

The incorporation of 5 wt% of sodium alginate polymer into the RC-BG resulted in a significant rise in the negative charge, especially at neutral and basic pH values. This behavior could be due to the carboxylic groups in this polymer, which progressively deprotonate to -COO^−^as the pH increases. The same trend was reported by El Baza et al., who investigated the effect of adding sodium alginate polymer on the surface charge of cement composed of DCPD and CaCO_3_, and attested that Alg increases the negative surface charge of the cement matrix (Elbaza et al., 2024). Similarly, Beraldo et al. (Beraldo et al., 2021) reported that sodium alginate exhibits increasingly negative zeta potential with rising pH, due to gradual carboxylate group ionization.

Additional results related to the setting reaction, setting time, injectability, and cohesion of the prepared cements are provided in the Supplementary Information (Figs. S3 and S4). These data are included to give a complete characterization of the formulations.

### 3.2 Adsorption study

#### 3.2.1 Kinetic Kineticstudy

**Fig. 5:**
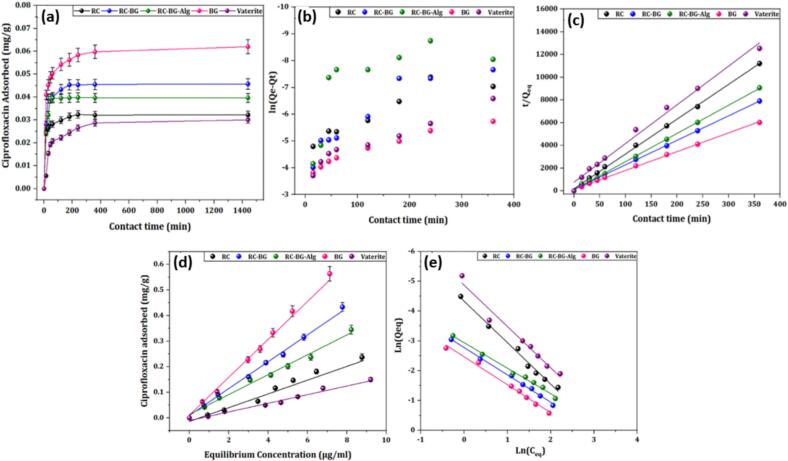
Ciprofloxacin uptake kinetics (a), the PFO kinetic (b), and the PSO kinetic (c) curves and ciprofloxacin amount adsorbed *vs* its equilibrium concentration: (d) Freundlich isotherms and (f) linearized logarithmic plots for the Cip adsorption onto Vaterite, BG, RC, RC-BG, and RC-BG-Alg.

The impact of contact time on the adsorption capacity of ciprofloxacin antibiotic molecules onto the surface of the investigated cements (RC, RC-BG, and RC-BG-Alg) is illustrated in Fig. 5.a. For comparison, adsorption experiments were also performed for bioactive glass (BG) and vaterite under the same conditions.

The kinetic study indicated a rapid uptake process for all the adsorbents under the examined conditions. Thus, the contact time required to establish the equilibrium was around 60 min. Comparable outcomes have been reported on the adsorption of the Cip antibiotic by several adsorbents, such as hydroxyapatite and hydroxyapatite-chitosan composite (Ait Said et al., 2023), biochar (Chakhtouna et al., 2022; Zeng et al., 2018), and natural phosphate (Bouyarmane et al., 2015). Moreover, it can be observed from the figure that the maximal adsorption capacity of these materials follows the following order: BG > RC-BG > RC-BG-Alg > RC > Vaterite*.* This difference will be explained in detail in the isotherm section.

To assess the potential mechanisms underlying ciprofloxacin uptake on the materials studied, two mathematical models were applied to examine the adsorption kinetics data (Ait Said et al., 2023; Wang et al., 2020).

The linear equations of pseudo-first-order (Eq. (11)) and pseudo-second-order kinetics (Eq. (12)) are described as:

(11) $$\ln\left(Q_{e}-Q_{t}\right)=\ln Q_{t}-k_{1}t$$

(12) $$\frac{t}{Q_{t}}=\frac{1}{k_{2}{Q_{e}}^{2}}+\frac{t}{Q_{e}}\;$$

where k_1_ (min^−1^) and k_2_ (g.mg^−1^.min^−1^) are the pseudo-first-order and the rate constants of pseudo-second-order model, respectively. The drug adsorbed quantities (mg.g^−1^) at a contact time t and equilibrium are respectively denoted by the letters Q_t_ and Q_e_.

When the pseudo-first-order model (PFO) is used, the obtained data showed non-linear plots for all samples (Fig. 5.b); the correlation coefficient values are low and range between 0.73 and 0.95 (Table 3). Whereas the best fit was given by the pseudo-second-order (PSO) with R^2^ values close to 1 (Fig. 5.c). The calculated amounts of the adsorbed antibiotic at equilibrium, using the PSO model, are well aligned with the experimental (Q_e_,_exp_) values. The low adsorption capacity and the good fit with the PSO kinetic imply that the adsorption process is likely dominated by chemisorption, with limited active site availability on the adsorbent surface. Similar observations were reported for the retention process of Cip by different materials such as natural phosphates (Bouyarmane et al., 2015; Zeng et al., 2018), multiwall carbon nanotube (Avcı et al., 2020), and biochar (Dou et al., 2022; Zeng et al., 2018), which were described by the PSO kinetic; the authors indicated that the interfacial phenomenon was controlled mainly by the chemisorption mechanism rather than the physisorption mechanism.

**Table 3:** Kinetic adsorption parameters determined by the PFO and PSO models. The adsorbed quantities Q_e,exp,_ and Q_e_ are given in mg/g^−1^.

Material	Q_e,exp_	PFO			PSO		
		Q_e_	K_1_ (min^−1^)	R^2^	Q_e_	K_2_ (g.mg^−1^ min^−1^)	R^2^
RC	0.033	6.5 10^−2^	1.70 10^−3^	0.77	0.033	5.02	0.99
RC-BG	0.046	1.0 10^−3^	2.40 10^−2^	0.87	0.046	3.40	0.99
RC-BG-Alg	0.040	1.0 10^−6^	2.12 10^−2^	0.73	0.040	6.88	0.99
BG	0.062	11.2 10^−3^	1.20 10^−2^	0.96	0.061	1.83	0.99
Vaterite	0.030	10.5 10^−3^	1.66 10^−2^	0.95	0.031	0.99	0.98

#### 3.2.2 Adsorption isotherm

Fig. 5.d and 5.e depict the amount of Cip adsorbed by RC, RC-BG, RC-BG-Alg, BG, and vaterite powders as a function of equilibrium concentration. It can be noticed that the adsorption profiles of the antibiotic were similar for all the cements. The adsorbed amount of the antibiotic varied linearly with its equilibrium concentration, suggesting that the process follows a Freundlich-type isotherm. To affirm this observation, the experimental data were fitted to the Freundlich equation (Eq. (13)). The adsorption parameters (a and m) were deduced from the linearized form of the Freundlich relation (Eq. (14)):

(13) $$Q_{\mathrm{ads}}=a.C_{\mathrm{eq}}^{m}\;$$

(14) $$\ln\left(Q_{\mathrm{ads}}\right)=\ln a+\mathrm{mln}C_{\mathrm{eq}}$$

where Q_ads_ (mg.g^−1^) denotes the amount of antibiotic adsorbed, and C_eq_ (mg.L^−1^) represents the concentration at equilibrium. The Freundlich constants “m” and “a” reflect the adsorption capacity and affinity, respectively.

The good coefficient correlation (*R*^*2*^ *close to 1) indicates that the adsorption data are well described by the Freundlich isotherm (*Table 4*), suggesting* a weak interaction between ciprofloxacin molecules and the matrix surface. Similar trends were reported in previous studies investigating the adsorption of simple drugs such as cisplatin (Barroug and Glimcher, 2002), gemcitabine hydrochloride and oxaliplatin (Betsiou et al., 2012), and serine (Benaziz et al., 2001) with calcium phosphate materials, as well as ciprofloxacin with biochar (Zeng et al., 2018) and hydroxyapatite-chitosan composites (Ait Said et al., 2023). These observations are generally attributed to the absence of high-affinity charged end groups, such as phosphonates and phosphates, and the fact that the adsorption process is primarily governed by polar functional groups (Cazalbou et al., 2015). In contrast, the adsorption process of other biologic and therapeutic compounds, including phosphoserine and amino acid molecules (Benaziz et al., 2001), or bisphosphonate (Errassifi et al., 2014; Pascaud et al., 2013) on calcium phosphate materials has been shown to be governed by the Langmuir-type isotherm, reflecting a strong affinity of these adsorbates for the apatite. This strong and specific affinity for the apatite surface is driven by the presence of phosphate or phosphonate groups and amino functionalities in the adsorbate, which can directly coordinate with calcium sites on the adsorbent.

**Table 4:** Adsorption parameters of Cip onto RC, RC-BG, RC-BG-Alg, BG, and vaterite in KCl.

Samples	Parameters		
	a (μL/g)	m	R^2^
RC	0.05	1.39	0.99
RC-BG	1.18	0.85	0.99
RC-BG-Alg	1.55	0.91	0.99
BG	3.47	0.94	0.99
Vaterite	0.02	1.38	0.99

The comparison of the obtained curves, as well as the association parameters, indicates that the incorporation of BG into the RC cement enhanced the uptake capacity, which increased from 0.24 mg/g for RC cement to 0.43 mg/g for RC-BG at an initial Cip concentration of 30 mg/L. However, the addition of the alginate polymer to the RC-BG specimen slightly reduced the adsorbed amount of Cip from 0.43 to 0.34 mg/g. The obtained values of the affinity constant (a) showed the following order: BG > RC-BG > RC-BG-Alg > RC > Vaterite*.* This disparity could be attributed to the modification of the cement's physicochemical properties (crystallinity index, surface area, surface functional groups, and porosity) after the incorporation of BG and Alg within the composite matrix, thereby influencing the reactivity and adsorption behavior of the final composite. The higher uptake capacity observed for BG can be attributed to its amorphous structure compared to the other composite compounds. Moreover, the lowest Cip uptake capacity of RC could be correlated to the high amount of vaterite phase introduced initially into the cement (50 wt%) compared to the other cements RC-BG and RC-BG-Alg, which contained 37.5 wt% of vaterite, since vaterite displayed the poorest affinity towards ciprofloxacin molecules.

Depending on the composition and surface charge of the adsorbents and the Cip ionization degree, several mechanisms, including electrostatic interactions, hydrogen bonding, and coordination bonding, can be involved at the adsorbent/adsorbate interface.

The degree of ionization of the functional groups in Cip molecules, as well as the surface charge of solid particles, may influence their adsorption on the carrier surface. The examined antibiotic has pKa values of 6.1 for the carboxylic acid and 8.7 for the basic amino groups (Bouyarmane et al., 2015; Mabroum et al., 2023). Accordingly, depending on the pH of the adsorption medium, Cip can take one of three forms: cationic, zwitterionic, or anionic. At a neutral pH, the most predominant species are zwitterionic and cationic. On the other hand, the surface charge of the powders is a further key factor influencing the adsorption process at the Cip/adsorbent interface. The examined cement powders had a negatively charged surface, as shown by the evolution of the zeta potential of the cement samples as a function of pH (Fig. 4), which likely hinders strong electrostatic interactions with zwitterionic Cip species.

Overall, the adsorption study revealed that the prepared specimens did not show significant retention capacity towards Cip molecules under the experimental conditions investigated. This indicates a low affinity between the antibiotic and the cement surface. Consequently, adsorption was not considered a viable method for incorporating Cip into the cement for drug delivery purposes. As an alternative strategy, the antibiotic was added to the liquid phase during the preparation of the Cip-loaded cement.

The low adsorption capacity of cements towards ciprofloxacin also demonstrates that the interaction of Ciprofloxacin with the cement systems does not significantly influence the antibiotic release, as other factors govern its release from the studied cements.

### 3.3 Drug release kinetics

**Fig. 6:**
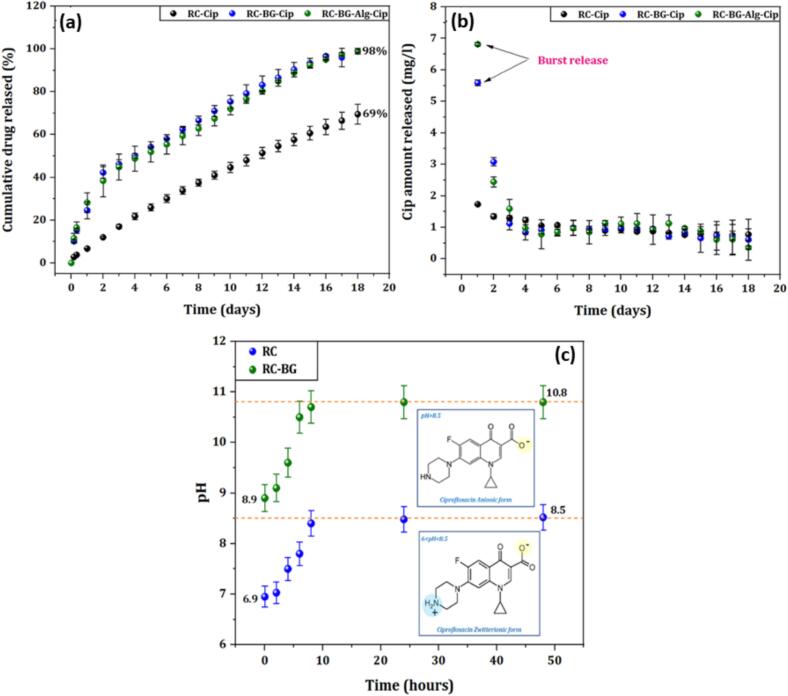
Cumulative Cip released rate (a) and amount per day of ciprofloxacin released in PBS solution (b) for ciprofloxacin-loaded cements, pH evolution during the setting of RC and RC-BG cements, and the forms of ciprofloxacin at different pH (c). The experiments were conducted in triplicate, and the results are presented as mean ± standard deviation (SD).

The effect of BG and Alg on the ability of the cements to release the active ingredient was evaluated in PBS solution at physiological pH for the cements loaded with 3 % antibiotic. The release kinetics of ciprofloxacin (expressed as a cumulative percentage) by RC-Cip, RC-BG-Cip, and RC-BG-Alg-Cip cements are presented in Fig. 6.a. At first sight, the obtained results indicate that the antibiotic elution occurred gradually throughout the investigated period, with a sustained release over 18 days. However, a massive release of Cip of about 25 % and 28 % was noticed during the first day, respectively, for RC-BG-Cip and RC-BG-Alg-Cip cements.

The evaluation of the cumulative liberation rate indicated that the percentage eluted was affected by adding BG and Alg within the matrix. Indeed, this rate increased significantly when bioactive glass 46S6 (BG) was incorporated into the RC cement matrix. After 18 days of release, the rate of ciprofloxacin eluted was 69 % for RC-Cip and 98 % for RC-BG-Cip, attesting that the latter specimen liberated almost all the antibiotic amount contained in the matrix. It is interesting to highlight that adding alginate to the RC-BG-Cip cement did not affect the rate of ciprofloxacin released after 18 days, which was maintained at about 98 % for the RC-BG-Alg-Cip composite cement.

The examination of the ciprofloxacin release quantities by the studied cements (Fig. 6.b) revealed that the quantities liberated each day (mg.L^−1^) were massive during the first two days of the release test. Indeed, on the first day, the RC-BG-Alg-Cip composite eluted 6.8 mg.L^−1^, followed by the RC-BG-Cip cement (5.6 mg.L^−1^). However, the RC-Cip cement released the lowest amount of ciprofloxacin, about 1.7 mg/L. The amount released tended to decrease for the remainder of the test, indicating that the released amounts on the second day were 1.3 mg/L for RC-Cip, 3.1 mg/L for RC-BG-Cip, and 2.4 mg/L for RC-BG-Alg-Cip. After the third day, the dose of active principle released was relatively constant, ranging between 0.36 and 1.59 mg/L. The amounts of the eluted drug are within the range of the minimum inhibitory concentration of Cip (0.25–2 mg.L^−1^) towards pathogenic germs responsible for bone infections, such as *S. epidermidis*, *S. aureus,* and *E. coli* (Castro et al., 2003; Venkatasubbu et al., 2011).

Several kinetic equations (Higuchi, Zero-order, and Korsmeyer-Peppas) were used to analyze the obtained release data to identify the mechanisms governing the release of Cip from the examined cements. The applicability of the Korsmeyer-Peppas model is limited to the initial 60 % of antibiotic release (Canal et al., 2013; Ritger and Peppas, 1987; Siepmann and Peppas, 2011). The linearized forms of the Korsmeyer-Peppas, zero-order, and Higuchi models were used to calculate the release kinetic parameters (Table 5).

**Table 5:** *In vitro* release kinetic constants of prepared cements.

Model	Parameters	RC-Cip	RC-BG-Cip	RC-BG-Alg-Cip
Zero-order	R^2^	0.992	0.934	0.945
	K_0_	0.155	0.189	0.186
Higuchi	R^2^	0.999	0.984	0.983
	K_H_	0.54	6.34	6.61
Korsmeyer - Peppas	R^2^	0.999	0.935	0.994
	n	0.72	0.49	0.43
	K_KP_	0.83	5.41	6.76

The regression coefficient (R^2^) revealed that the Higuchi equation provided the best fit for the kinetic release of ciprofloxacin from all cements, as the curves showed maximum linearity, followed by the Korsmeyer-Peppas model. This explains the phenomenon known as square root kinetics, which states that the drug diffuses more slowly as the distance for diffusion rises (or Higuchi's kinetics). However, the drug release from RC-Cip was also found to fit very well with zero-order kinetics. This suggests that the dissolution and release of the drug occur solely depending on time and happen regardless of the drug concentration at any specific moment. In particular, zero-order kinetics can be used to outline the release of poorly soluble active species contained in a matrix system (Varelas et al., 1995), which is the case for RC-Cip cement. Indeed, the solubility of Cip is strongly dependent on the pH (Roca Jalil et al., 2015). For a neutral pH, as was the case with RC-Cip cement (the pH of the RC-Cip cement paste was found to be around 7 to 8), Cip had a low solubility. This low solubility explains the slow release of the drug from this cement, a process that closely resembles zero-order kinetics. For this cement (RC-Cip), the coexistence of Higuchi and zero-order kinetics in drug release from RC-Cip suggests a complex release mechanism. It implies that while the rate of drug diffusion decreases with increasing time (square root kinetics), the overall release of the drug from the system remains constant (zero-order kinetics).

The RC-BG-Cip and RC-BG-Alg-Cip cements best fitted the Higuchi and Korsmeyer-Peppas models, suggesting that the elution mechanisms of ciprofloxacin involve diffusion-controlled release processes (Higuchi kinetics) and a possible combination of diffusion and other phenomena, such as erosion (Korsmeyer-Peppas).

To further investigate the mechanisms governing the ciprofloxacin release from the RC-BG-Cip and RC-BG-Alg-Cip cements, the diffusional exponent (n) of the Korsmeyer-Peppas equation was investigated.

In the case of RC-Cip cement, the Korsmeyer-Peppas equation showed a value of the exponent “n” of 0.72 (0.45 < *n* < 1), revealing that the elution of Cip occurred in an atypical manner and that other mechanisms could also contribute to the release of the drug. Based on the shape of the release curve, the low solubility of Cip, and the value of the coefficient “n” located between the two limit values (0.45 indicating Fick's law, and 1 indicating zero-order kinetics), it can be concluded that the mechanisms involved in this case are probably related to the coexistence of these two diffusion phenomena: diffusion according to Fick's law and zero-order kinetics.

Moreover, the exponent “n” values of the cements RC-BG-Cip (*n* = 0.49) and RC-BG-Alg-Cip (*n* = 0.43) are different, suggesting that the processes driving the ciprofloxacin release kinetics may be different. The RC-BG-Cip cement showed a value of n = 0.49, indicating that, in addition to the diffusion, different processes contribute to drug release. The cement RC-BG-Alg-Cip exhibited a value of the exponent of 0.43, which attests that the antibiotic elution follows Fick's law.

A common strategy used to optimize drug release from cement matrices is to modify the liquid and solid phase composition of the cement formulation. The physicochemical properties of the CPCs, such as microstructure, composition, porosity, *etc.*, are typically affected by the modification of these phases. Therefore, these parameters can influence the release behavior of the active ingredients.

Generally, incorporating additives into CPC can also impact the drug affinity for the inner matrix surface by altering the surface charge and pH variations. Depending on the type and amount of additive introduced, the CPC / drug binding strength can be strengthened or weakened, leading to alterations in the release kinetics. When a biodegradable polymer is added, its rapid degradation accelerates the release kinetics compared to the CPC, and this may result in a massive release of the drug (Fosca et al., 2022). The chemical structure of the drug also directly affects the release rate.

In this work, porosity did not appear to be a major factor responsible for the increased antibiotic release. Indeed, adding BG to the RC cement increased the released rate of ciprofloxacin from 69 % to 98 %, while the porosity values are 51.6 % and 48.0 % for RC and RC-BG cements, respectively (Table 2). Furthermore, the Cip adsorption tests performed on the RC and RC-BG cement powders revealed low retention capacity, pointing out weak attraction forces between this antibiotic and the surface of the cements studied. This suggested that Cip elution was governed neither by porosity nor by the interaction between this antibiotic and the surface of the cements, but could be related to other parameters, such as the pH of the cement/release medium, the solubility of the antibiotic, as well as the surface charge of the cements. As ciprofloxacin is mixed with the powder phase of the cements, the mixture undergoes a setting reaction and, consequently, a change in pH, leading to a change in the charge of ciprofloxacin and, consequently, its solubility.

It is commonly recognized that the solubility of ciprofloxacin (Cip) impacts the pH of the medium. Indeed, Cip is highly soluble in water under high pH levels (pH ≥ 9) but poorly soluble at pH values ranging between 6 ≤ pH ≤ 8 (Roca Jalil et al., 2015). Roca et al. plotted an experimental solubility curve of Cip from different saturated Cip solutions that were pH-adjusted to range from 4 to 11 (Roca Jalil et al., 2015). The pH range between 7 and 7.5 yields the lowest solubility values. The species found in each pH range can explain Cip's solubility behavior as pH varies. Soluble Cip^+^ species are present at the lowest pH values, and their percentage decreases as the pH rises from 3 to 5.9. Three different species are found in the pH range of 5.9 (pKa1) to 8.9 (pKa2); the zwitterionic form of Cip, which has a neutral charge, is the least soluble and reaches its lowest solubility in water at a pH of 7.5. Due to the presence of more soluble Cip-species, which reached 50 % at pH 8.9 and increased pH values above 8.9, Cip became more soluble with increasing pH.

The measurement of the pH of the cement pastes (Fig. 6.c) showed that the combination of bioactive glass 46S6 with the reference cement RC increased the pH of the prepared cement paste from 6.9 for CPC to 8.9 for the CPC-BG cement paste. After 48 h of maturation and hardening, the pH of the cement prepared with BG (RC-BG) increased to a value of pH = 10.8. In this pH condition, the carboxyl group of ciprofloxacin became negatively charged, thus inducing an increase in the solubility of the antibiotic and, consequently, its rapid elution.

The slow-release rate observed for the reference cement could be due to the pH of the cement paste (pH = 6.9–8.5), which favors the presence of the zwitterionic form of Cip in this pH range, where its solubility is low. The surface charge of the cement materials could also explain the difference in the release behavior. As demonstrated by the zeta potential measurements, the addition of BG and Alg to the RC cement dramatically increases the negative surface charge. The increased negativity of the surface charge related to the negatively charged groups in BG and Alg in the cement compositions could induce repulsive interactions towards ciprofloxacin, which was in anionic form in the pH range of 8.9 to 11, resulting in increased release of the antibiotic, thus, a massive release occurred on the first day of the release test. It is well known that BGs have intrinsic porosity and a strong tendency to degrade in solution by producing hydrated silica species (Schumacher et al., 2021). Theoretically, this factor ought to increase the release rate of the active species. In addition, the silicate group in the calcium phosphate network increased the surface charge negativity (Vallet-Regí et al., 2003) due to the formation of Si-OH groups on the surface. This, in turn, accelerated the rate of Si ions dissolution (Arcos et al., 2003), leading to a faster drug release*.*

Jani et al. designed a composite consisting of pure mesoporous silica and hydroxyapatite as a release system for the ibuprofen molecule (IBU). The delivery behavior of this composite was compared to that of the mesoporous silica (MCM) compound (Jani et al., 2020). According to the findings, the amount of drug released by the MCM compound was more than that released by the composite. The mesoporous silica material also demonstrated a higher release rate, resulting in a total release of the embedded drug within the first 10 h. The composite displayed a minor massive effect in the early hours, followed by a sustained release behavior. The massive release observed for the silica material can be attributed to its high degradation rate compared to hydroxyapatite. The IBU molecule possessed carboxyl groups, which might interact with the Ca^2+^ ions of the composite, reducing the mobility of the IBU within the matrix and consequently slowing down the release from the composite system.

The use of alginate seemed not to affect the released rate of the active ingredient but tended to increase the amount released during the first day from a value of 5.6 mg/L for the RC-BG-Cip cement to a value of about 6.8 mg/L for the RC-BG-Alg-Cip composite. This effect could be due to the degradation of the polymer on the surface of the cement due to its high solubility in the PBS medium. The use of alginate as a biodegradable polymer whose degradation is expected to produce vacant cavities in the composite cement, thus opening channels for faster release of ciprofloxacin. The electrostatic repulsions between the alginate's negatively charged carboxylic groups and those of Cip could also explain the burst release of the antibiotic during the first day.

On the contrary, David Chen et al. demonstrated that the addition of 0.5 % alginate to a CPC composed of CaCO_3_ and MCPM greatly decreased the gentamycin release rate from 70 % over the first 24 h in the free-alginate cement to 51 % for the cement containing alginate (Chen et al., 2011). This effect is the result of the interaction of gentamycin with the alginate.

### 3.4 Post-release characterization

#### 3.4.1 Composition and microstructure

**Fig. 7:**
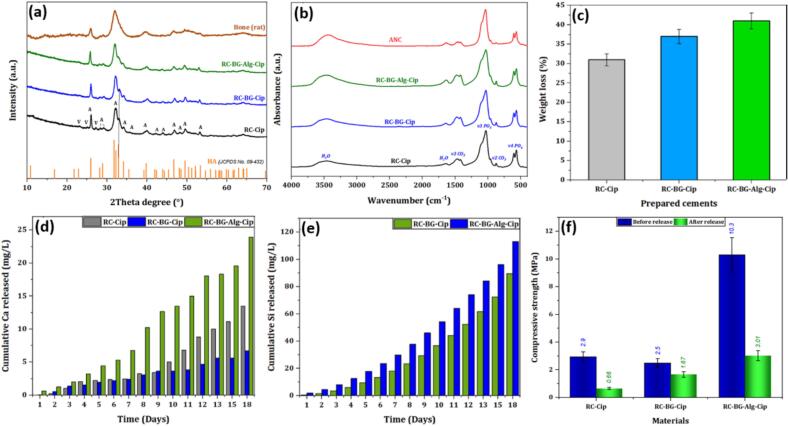
Post-release XRD diagrams (a) and FTIR spectra (b) of RC-Cip, RC-BG-Cip, and RC-BG-Alg-Cip cements compared to Hydroxyapatite (HA), bone rate, and nanocrystalline carbonated apatite NCA, weight loss percentage after 18 days of immersion in PBS medium (c), cumulative released concentrations of Ca (d) and Si (e) from RC-Cip, RC-BG-Cip, and RC-BG-Alg-Cip cements. (f) compressive strength of RC-Cip, RC-BG-Cip, and RC-BG-Alg-Cip cements before and after 18 days of immersion in PBS medium.

Fig. 7.a depicts the XRD patterns of post-release cements (RC-Cip, RC-BG-Cip, and RC-BG-Alg-Cip). All cements demonstrated changes in phase composition after 18 days of immersion in PBS. The diffractograms of RC-BG-Cip and RC-BG-Alg-Cip demonstrated the presence of a poorly crystalline apatite, similar to the mineral part of the bone. The vaterite peaks were not detected in the diffractograms of these cements. This could be due to the dissolution of the vaterite phase or its transformation into the apatite phase in the presence of the PBS solution medium, which is rich in phosphate ions, thereby promoting apatite formation. In contrast, the RC-Cip diagram showed some vaterite peaks with low intensity (at 25.1° and 27.3°), indicating the presence of traces of vaterite in addition to apatite.

Fig. 7.b illustrates the FTIR spectra of the post-release cements (RC-Cip, RC-BG-Cip, and RC-BG-Alg-Cip) compared to the FTIR spectrum of nanocrystalline carbonated apatite (NCA). All the FTIR spectra appeared similar, testifying to the characteristic bands of phosphate groups between 500 and 750 cm^-1^ and 1000 and 1250 cm^-1^, as well as carbonate groups around 900 cm^-1^ related to the ν2 CO_3_ and ν3 CO_3_ vibrations in the region between 1400 and 1550 cm^−1^. No ciprofloxacin bands were observed, consistent with drug release. Comparing the FTIR spectra of the cements and the nanocrystalline carbonated apatite (NCA), a difference in the carbonate intensity band between 1400 and 1550 cm^−1^ is observed, which could be due to the overlap of the NCA band and the vaterite traces bands. These results confirmed those obtained in the XRD analysis, revealing the precipitation of a nanocrystalline carbonated apatite phase.

#### 3.4.2 Degradation in PBS medium

After 18 days of the ciprofloxacin release test, the cement's degradation rate was evaluated based on their weight loss ratios in PBS medium (Fig. 7.c).

The results revealed that the degradation rate of RC, which was composed solely of apatite and CaCO_3_, was slower compared to that of cements containing BG. Indeed, the degradation rate increased from 31 % to 37 % for RC-Cip and RC-BG-Cip, respectively. Moreover, the addition of Alg gel to the cement resulted in a 41 % increase in degradation rate, due to the rapid dissolution of BG and degradation of alginate.

The degradation of the cements was also evaluated by measuring the concentration of calcium (Ca) and silica (Si) released in the PBS medium. Figs. 7.d and 7.e depict the released concentration of Si and Ca elements from the RC-Cip, RC-BG-Cip, and RC-BG-Alg-Cip cements. The results showed that for all cements, the concentration of ions released increased progressively with increasing soaking duration. Furthermore, the release kinetics of Si in solutions were generally faster compared to those of *Ca.* The cumulative amounts of Ca released after 18 days are 13, 7, and 24 mg/L for RC-Cip, RC-BG-Cip, and RC-BG-Alg-Cip, respectively. Moreover, the cumulative quantities of Si released after 18 days are 90 and 113 mg/L for RC-BG-Cip and RC-BG-Alg-Cip cements, respectively. The difference in released concentrations between Ca and Si observed for RC-BG-Cip and RC-BG-Alg-Cip cements is due to the higher solubility of glass (the source of Si) compared to apatite (the source of calcium ions). During the release test, the calcium ions released from the vaterite interacted with the phosphate ions originating from the PBS and bioactive glass-forming apatite in the cement. The high solubility of Alg increased the degradation of the cement composite RC-BG-Alg-Cip, resulting in higher concentrations of Ca and Si released.

The mechanical performance before and after immersion was also evaluated through compressive strength measurements (Fig. 7.f). Before immersion, RC-Cip and RC-BG-Cip exhibited compressive strengths of 2.9 and 2.5 MPa, respectively, while RC-BG-Alg-Cip reached 10.3 MPa, highlighting the reinforcing effect of alginate in the BG-containing formulation. After 18 days of immersion, all cements showed a significant reduction in strength, with RC-Cip, RC-BG-Cip, and RC-BG-Alg-Cip retaining only 0.86, 1.67, and 3.07 MPa, respectively. This decrease is consistent with the observed weight loss and ion release trends, confirming that prolonged immersion and matrix dissolution lead to a marked loss of mechanical integrity, particularly in formulations with higher degradation rates.

### 3.5 Antibacterial activity

**Fig. 8:**
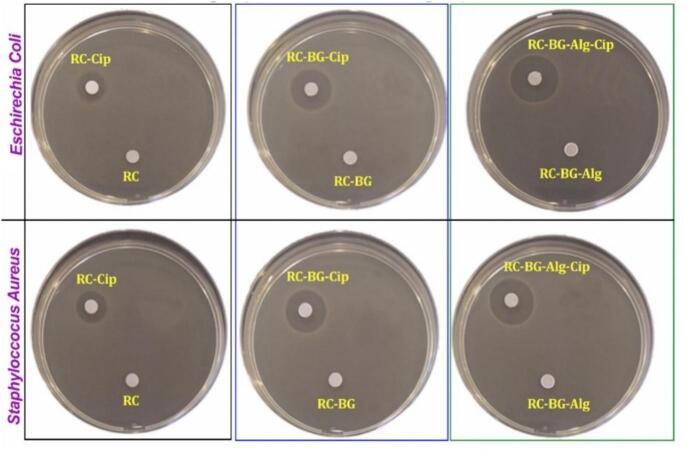
Antibacterial activity of cements without Cip (RC, RC-BG-Alg, and RC-BG) and cements containing Cip (RC-Cip, RC-BG-Cip, and RC-BG-Alg-Cip) against *E. coli* and *S. aureus* bacteria.

To examine the impact of bioactive glass, alginate, and ciprofloxacin on antibacterial activity towards *E. coli (EC)* and *S. aureus (SA)*, cements were evaluated with and without ciprofloxacin (Fig. 8). The inhibitory zone diameter data are summarized in Table 6. The antibiotic-free cement (RC, RC-BG, and RC-BG-Alg) had no antibacterial action against *EC* and *SA* strains, according to the findings (there was no inhibition zone visible surrounding the discs). Cements containing ciprofloxacin significantly reduced the growth of the bacteria around the discs. Incorporating 3 % ciprofloxacin in the reference cement (RC-Cip) resulted in an inhibition diameter of 14.0 mm for both *S. aureus* and *E. coli*. For RC-BG-Cip cement, the inhibition zone diameter was found to be 18.5 mm and 19 mm for *SA* and *EC,* respectively. The composite cement RC-BG-Alg-Cip had the highest inhibition zone diameter against both germs. Furthermore, adding bioactive glass and alginate polymer significantly improved the inhibitory zone diameter for both germs. As confirmed by the release test, the increase in the inhibitory zone diameter could be attributed to the increased antibiotic concentrations released from the examined cements. The antibacterial activity of the formulated composites in inhibiting the growth of *EC* and *SA* is owing to the therapeutic amounts of antibiotic released daily, which range from 0.25 to 2 μg/mL and are near the minimum inhibitory concentration (MIC) of ciprofloxacin (Raj et al., 2013).

**Table 6:** Inhibition zone diameters of cements without Cip (RC, RC-BG, and RC-BG-Alg) and cements containing Cip (RC-Cip, RC-BG-Cip, and RC-BG-Alg-Cip) against the selected germs (*E. coli* and *S. aureus)*.

Cements	Inhibition zone diameter (mm)	
	***E. coli***	***S. aureus***
RC	ND*	ND*
RC-BG	ND*	ND*
RC-BG-Alg	ND*	ND*
RC-Cip	14.0	14.0
RC-BG-Cip	18.5	19.0
RC-BG-Alg-Cip	21.5	20.0

ND*: Not detected.

### 3.6 *In vitro* cytotoxicity: Cell Viability – MTT assay

**Fig. 9:**
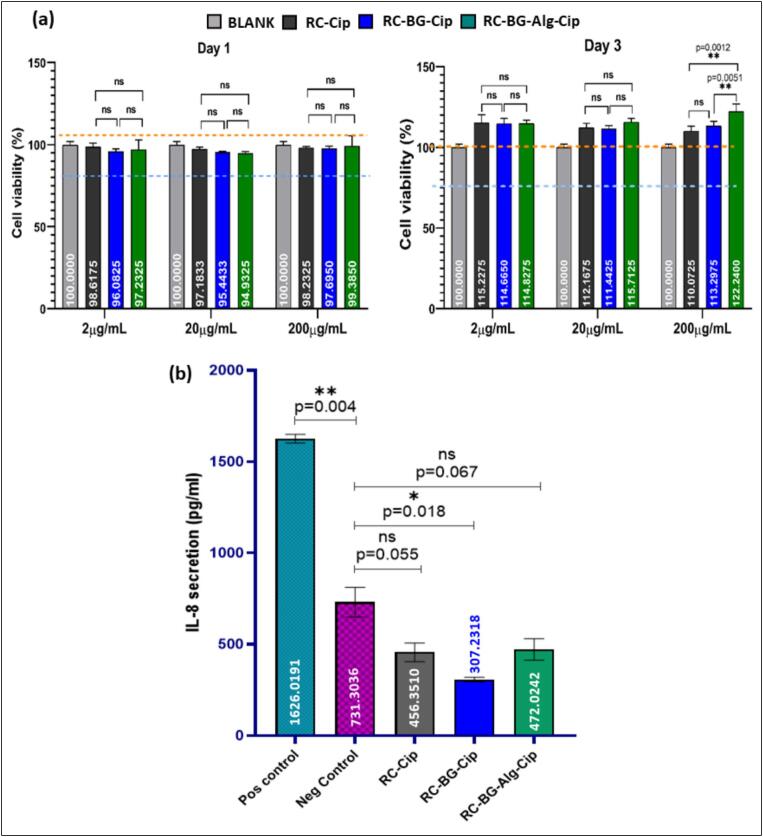
(a) *In-vitro* cytotoxicity test of the RC-Cip, RC-BG-Cip, and RC-BG-Alg-Cip cements at different concentrations (2 μg/mL, 20 μg/mL, and 200 μg/mL) incubated for 1 and 3 days, and (b) the quantification of IL-8 secretion in the supernatant of hPBMCs after their treatment with the different cement types at a concentration of 200 μg/mL after 8 h of incubation. The LPS was used as a positive control. The bars represent the SD values for each measurement, which were performed in duplicate. The graphs represent the mean ± SD values.

The cytotoxicity effect of different concentrations of the ciprofloxacin-loaded cements on hPBMCs was first analyzed. The results showed that, independently of the concentration, the ciprofloxacin-loaded cements did not affect the cell viability, with percentages close to 100 % and no significant differences compared with the negative control (Fig. 9.a).

Notably, minor variations in cell viability were observed at high concentrations, with no significant difference, particularly in the RC-BG-Alg-Cip group at the highest concentration (200 μg/mL), where a slight reduction in cell viability was observed. These results imply that the initial interaction of cells with the cement materials is beneficial, supporting their biocompatibility (Fig. 9.a).

Extending the incubation period to three days, the results show an increase in cell viability for all cements, with values exceeding the negative control, indicating that prolonged exposure to these materials does not compromise cell health. At a concentration of 200 μg/mL, RC-BG-Alg-Cip cement had significantly higher cell viability (122.24 %) than RC-BG-Cip (113.29 %), and RC-Cip cement, which showed a slight decrease in cell viability when increasing the concentration from 2 to 200 μg/mL with percentages of 115.22 %, 112.16 %, and 110.07 %, respectively. This increase in cell viability suggests that, in addition to being non-cytotoxic, the RC-BG-Alg-Cip formulation may possess properties that promote cell proliferation, potentially due to the bioactivity of its components, such as calcium phosphate, bioactive glass, and alginate, which are known to support cell attachment and growth. For instance, the ions released, such as calcium and phosphorus (Li et al., 2008; Samavedi et al., 2013), as well as silicate (Han et al., 2013), have been shown to improve the biological response, including cell proliferation. This hypothesis is supported by the biodegradation results, which revealed that after three days of incubation, the addition of BG and Alg resulted in increased Ca and P concentrations released, with higher values for the RC-BG-Alg-Cip. Moreover, the release of Si ions from cements like RC-BG-Cip and RC-BG-Alg-Cip could promote cell proliferation, leading to an enhancement in cell viability compared to our reference cement (RC-Cip). Recent studies have highlighted the advantages of incorporating bioactive glass in calcium phosphate cements (CPC) to improve their biological properties (Demir-Oğuz et al., 2023). For instance, Yu et al. reported that incorporating BG (45S5) into CPC, which is composed of TTCP and DCPA, significantly enhanced cell growth and proliferation, showing no cytotoxic effects on cells compared to the CPC reference cement (Yu et al., 2013). Similarly, Sadiasa et al. investigated the effect of adding BG into the CPC cement composed of TTCP and DCPD (Sadiasa et al., 2014). Their findings showed that increasing the concentration of bioactive glass enhances cell proliferation. These studies corroborate our results, suggesting that the enhanced cell viability observed in RC-BG-Cip and RC-BG-Alg-Cip formulations is likely due to the synergistic effects of the released ions and the bioactivity of the incorporated glass. As a result, incorporating bioactive glass into CPC appears to be an intriguing approach for enhancing the biological performance of these materials, making them suitable candidates for bone regeneration and other orthopedic applications.

### 3.7 Effect of ciprofloxacin-loaded cements on inflammatory response

To investigate whether ciprofloxacin-loaded cements have any effect on hPBMCs mediating inflammation, we measured the IL-8 release after incubating hPBMCs with 200 μg/mL of different cement formulations for 8 h. Lipopolysaccharide (LPS) is used as a positive control. The ELISA results showed that the pre-treatment of the hPBMCs with different cement formulations did not influence the inflammatory response, as there were no noteworthy variations in the IL-8 release between the negative control (hPBMCs incubated only with culture medium) and the hPBMCs pre-treated with cements (Fig. 9.b).

The results highlighted that the cements had a non-inflammatory effect when investigating the induction of IL-8 secretion, suggesting that there was no activation of immune cells. The IL-8 secretion comparison between the negative control (731.3 pg/mL) and the different cements showed a decrease in cytokine release. The presence of ciprofloxacin antibiotic in the cement composition could explain this reduction. In line with this finding, fluoroquinolones have been reported to confer immunomodulatory actions on a wide range of cell types (Dalhoff, 2005). For instance, F. Sachse et al. demonstrated that the ciprofloxacin antibiotic has anti-inflammatory properties on the IL-8 synthesis in an *in vitro* study of *S. aureus* Newman-induced nasal inflammation (Sachse et al., 2008).

In comparing the IL-8 secretion levels among the three investigated cements, the results showed a decrease in IL-8 secretion from 453.3 pg/mL to a value of 307.2 pg/mL when BG was incorporated into the RC-Cip cement. However, when sodium alginate was added (RC-BG-Alg-Cip), the IL-8 secretion increased to 472.0 pg/mL, which was not different from the negative control. This finding suggests a potential link to the ciprofloxacin concentrations released in the medium. The results of the ciprofloxacin release kinetics revealed that adding BG to the RC-Cip matrix significantly increased the amount of ciprofloxacin released, which may explain the decrease in IL-8 secretion observed in the cement RC-BG-Cip. Furthermore, it has been reported that BG could influence the inflammatory response, which could depend on its characteristics, particularly the composition and dose used, as well as the immune cells implicated (Zheng et al., 2021). For instance, the first prepared 45S5 BG was found to downregulate the release of pro-inflammatory TNF-α and IL-6 cytokines in activated macrophages at a dosage of 0.031 mg/cm^2^ (Day and Boccaccini, 2005).

The RC-BG-Alg-Cip cement, which combines ciprofloxacin, bioactive glass, and alginate, showed a mean IL-8 secretion of 472.02 pg/mL. Despite that, this cement exhibited an extended-release dose of ciprofloxacin; the IL-8 levels increased slightly compared to the RC-BG-Cip formulation, but still below the negative control. This observation raises questions about the potential involvement of alginate in increasing the IL-8 release in the case of RC-BG-Alg-Cip compared to RC-BG-Cip.

The *in vitro* biological investigation performed in this work demonstrated the nontoxicity of the ciprofloxacin-loaded cements, even after a three-day incubation period. These findings suggest the notable therapeutic potential of these cements, thereby paving the way for *in vivo* investigation using animals. Furthermore, the designed cement loaded with ciprofloxacin exhibited a decrease in IL-8 secretion, suggesting a non-inflammatory response. The antibacterial, non-cytotoxic, and anti-inflammatory properties of the cements are promising signs of the safety and relevance of these self-setting materials in therapeutic applications, such as drug delivery and bone substitutes, for preventing and treating related bone-implant infections.

## 4 Conclusion and perspectives

The main objective of this study is to explore the effect of BG and Alg on the key factors influencing the adsorption and release of Cip from an antibacterial composite cement prepared from DCPD, CaCO_3_, sodium alginate (Alg), bioactive glass (BG), and ciprofloxacin (Cip). These factors include the pH, SSA, porosity, surface charge, solubility of Cip, and the interaction between Cip and the cement matrix. The adsorption tests revealed that the retention capacity depends on the degree of ionization of Cip molecules' functional groups, as well as the surface charge of solid particles. However, low retention capacities are noticed for all formulations tested, confirming the weak interactions between Cip and the cement surfaces. The release tests showed that the addition of BG and Alg into reference cement increased Cip release from 69 % to 98 %, even though porosity decreased slightly from 51.6 % to 48.0 %. This suggests that Cip release was not governed by porosity or adsorption, but rather by other parameters altered by BG and Alg, such as the pH of cement paste and drug solubility. The antibacterial tests of the prepared cements against *E. coli* and *S. aureus* germs attested to their effectiveness in eradicating these germs. Furthermore, Alg and BG improved the cement's degradability and cell viability without causing any cytotoxic or inflammatory effects, confirming their *in vitro* bioactivity and biocompatibility. Despite these promising results, this study focused on *in vitro* assessments under simplified conditions, which may not accurately reflect the complexity of the *in vivo* environment. Antibacterial testing was limited to two bacterial strains, and cytotoxicity/inflammatory evaluations were performed in a short-term setting, with a focus on hPBMCs. Further research should focus on long-term degradation and release under physiologically relevant conditions, broaden the antibacterial range, and conduct *in vivo* experiments to validate the obtained *in vitro* results and the potential of ciprofloxacin-loaded cements as drug carriers for targeted treatment of bone infections.

## CRediT authorship contribution statement

**Hanaa Mabroum:** Writing – original draft, Methodology, Investigation, Formal analysis, Conceptualization. **Hamid Ait Said:** Writing – review & editing, Formal analysis. **Hamza Elbaza:** Writing – review & editing, Investigation, Formal analysis. **Yousra Hamdan:** Writing – review & editing, Investigation, Formal analysis. **Said Zayane:** Writing – review & editing. **Rachid Hakkou:** Resources. **Sanae Ben Mkaddem:** Writing – review & editing, Resources. **Rachid El Fatimy:** Writing – review & editing, Resources. **Hicham Ben Youcef:** Writing – review & editing, Resources, Methodology. **Hassane Oudadesse:** Writing – review & editing, Supervision. **Hassan Noukrati:** Writing – review & editing, Supervision, Methodology, Funding acquisition, Conceptualization. **Allal Barroug:** Writing – review & editing, Supervision, Project administration, Methodology, Funding acquisition.

## Declaration of competing interest

The authors declare that they have no known competing financial interests or personal relationships that could have appeared to influence the work reported in this paper.

## Data Availability

Data will be made available on request.
