# Supplementary material for: Synergistic effects of bioactive glass and sodium alginate on the surface properties and therapeutic release of ciprofloxacin from apatite cements
Source: Int J Pharm X. 2025 Sep 19;10:100401. doi: 10.1016/j.ijpx.2025.100401 (PMC12508814; doi:10.1016/j.ijpx.2025.100401)
Supplement: Supplementary file 1 — Supplementary material [file mmc1.docx]

***Supplementary information***

**Synergistic Effects of Bioactive Glass and Sodium Alginate on the Surface Properties and Therapeutic Release of Ciprofloxacin from Apatite Cements**

**Hanaa Mabroum ^a *^, Hamid Ait Said ^b^, Hamza Elbaza ^a^,** **Yousra Hamdan ^a^, Said Zayane^a^, Rachid Hakkou ^c,d^**, **Sanae Ben Mkaddem ^a^,** **Rachid El Fatimy ^a^,** **Hicham Ben Youcef ^b^, Hassane Oudadesse ^e^, Hassan Noukrati ^a *^, Allal Barroug ^a,f^**

*^a^ Faculty of Medical Sciences, UM6P Hospitals, Mohammed VI Polytechnic University, Benguerir, 43150, Morocco,*

*^b^ High Throughput Multidisciplinary Research Laboratory (HTMR), Mohammed VI Polytechnic University (UM6P), Benguerir, Morocco,*

*^c^ Laboratory of Innovative Materials, Energy and Sustainable Development (IMED-Lab), Faculty of Science and Technology Gueliz, Cadi Ayyad University (UCA), Marrakech, Morocco*

*^d^ Geology and Sustainable Mining Institute (GSMI), Mohammed VI Polytechnic University (UM6P), Benguerir, Morocco*

*^e^ Univ Rennes, CNRS, ISCR-UMR 6226, F-35000 Rennes, France*

*^f^ Cadi Ayyad University, Faculty of Sciences Semlalia, 2390, 40000, Marrakech, Morocco*

*** Corresponding Author**

**Hassan NOUKRATI, Assistant Professor**

Email: [hassan.noukrati@um6p.ma](mailto:hassan.noukrati@um6p.ma)

Mohammed VI Polytechnic University (UM6P), Faculty of Medical Sciences (FMS), UM6P Hospitals, Hay Moulay Rachid 43150, Benguerir, Morocco

**Hanaa MABROUM, Postdoctoral researcher**

Email: Hanaa.MABROUM-EXT@um6p.ma

Mohammed VI Polytechnic University (UM6P), Faculty of Medical Sciences (FMS), UM6P Hospitals, Hay Moulay Rachid 43150, Benguerir, Morocco

1. ***Particle size distribution of BG***

The particle size distribution of the bioactive glass 46S6 used in this study was determined by laser diffraction (**Figure S1**). The distribution is monomodal with a median particle size of 198 ± 8 μm. The particle-size distribution parameters attesting that 10% of particles by volume were smaller than 131 ± 5 μm, 50% were smaller than 198 ± 8 μm, and 90% were smaller than 295 ± 12 μm.


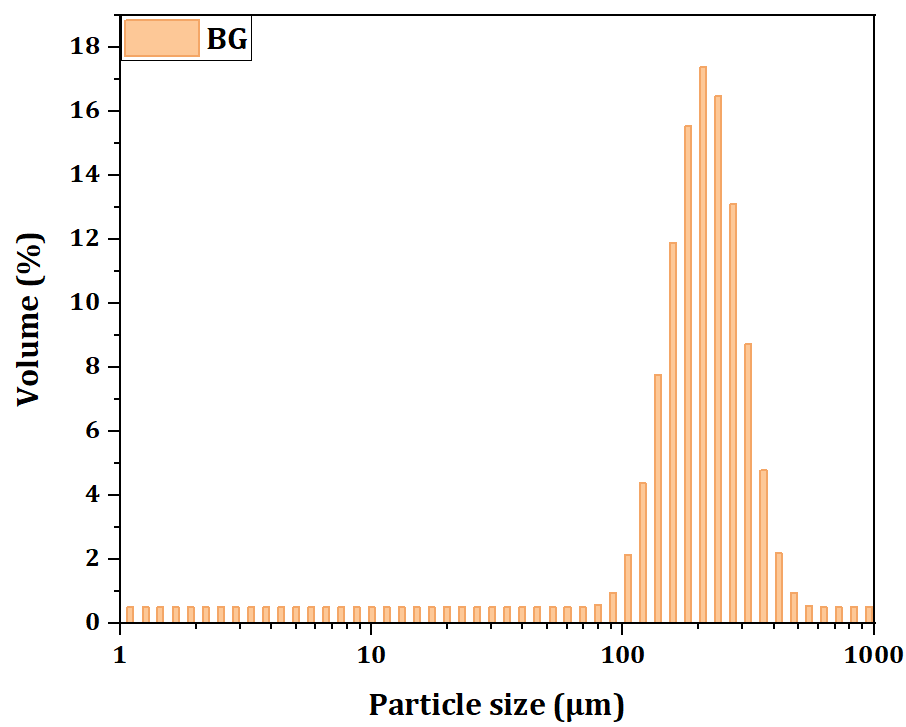


**Figure S1.** Particle size distribution of bioactive glass 46S6.

**2- Calibration curve of ciprofloxacin**

Ciprofloxacin quantification was carried out using UV-Vis spectrophotometry at 271 nm. Calibration curves were freshly prepared for each set of measurements and consistently showed high linearity (R^2^ > 0.99), ensuring accurate quantification within the tested range (Figure S2).

**Figure S2.** Calibration curve of ciprofloxacin in PBS (pH 7.4) measured by UV-Vis spectrophotometry at 271 nm.

***3- Cements properties***

- 1. ***Setting reaction***

The effect of bioactive glass (BG) and sodium alginate (Alg) on the setting reaction kinetics of ciprofloxacin-loaded reference cement (RC-Cip) was investigated. Fresh pastes were incubated for different time intervals (2, 4, 6, 8, 24, and 48 h) under 100% relative humidity at 37 °C and subsequently freeze-dried to halt the reaction. The structural evolution was then monitored by XRD (10°–60°), focusing on the transformation of brushite (DCPD) into apatite phases (Figure S3).

As shown in Figure S3.a, the XRD patterns of RC-Cip revealed intense DCPD peaks at ~11.7°, 23.5°, and 29.3°, which gradually decreased and disappeared completely after 48 h. Concurrently, apatite reflections appeared after 8 h, confirming the typical setting reaction of brushite-based cements. Incorporation of BG significantly accelerated this transformation (Figure S3.b): DCPD reflections disappeared after only 8 h, while apatite peaks were already visible at 4 h. This acceleration can be attributed to the release of Ca²⁺ and silicate ions from BG, which enhance supersaturation and promote apatite nucleation and growth.

An even greater acceleration was observed in the RC-BG-Alg-Cip composite (Figure S3.c), where DCPD peaks were no longer detected after 6 h, indicating complete consumption of brushite within a shorter period. This pronounced effect likely arises from the synergistic action of BG and Alg. In addition to ionic release from BG, sodium alginate contributes by increasing the pH and modifying the ionic environment of the paste, thereby creating more favorable conditions for apatite precipitation and growth.


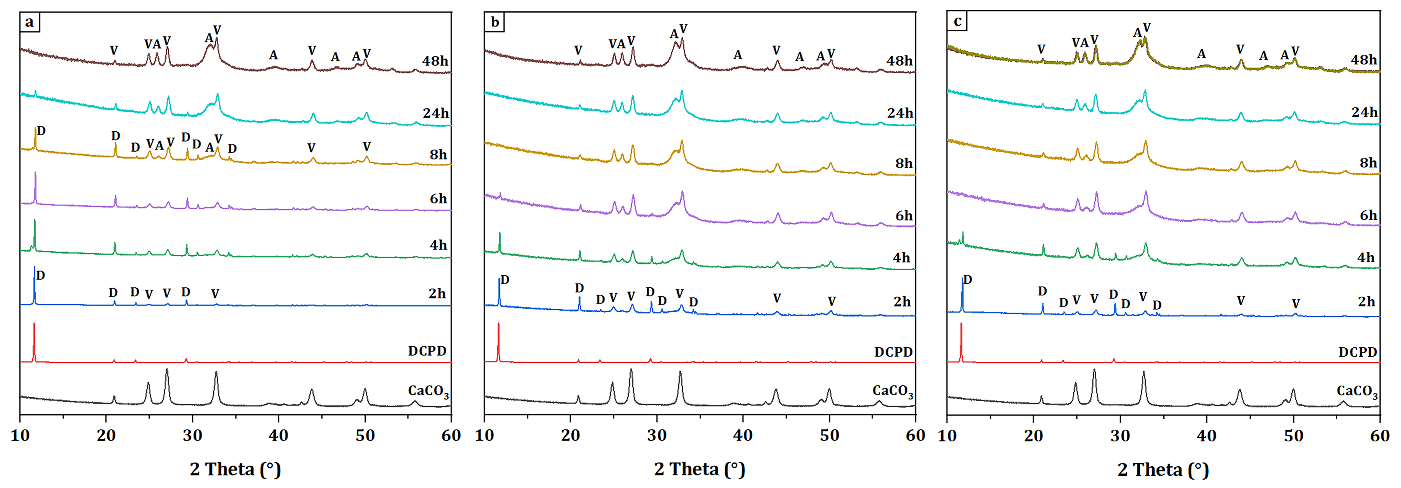


**Figure S3.** XRD patterns of RC-Cip (a), RC-BG-Cip (b), and RC-BG-Alg-Cip (c) cements at 2, 4, 6, 8, 24, and 48 hours of setting reaction (D: DCPD, V: Vaterite, and A: Apatite).

- 1. ***Setting time, compressive strength, injectability, and cohesion***

The initial and final setting times of the specimens were determined using a Gillmore needle apparatus in accordance with ASTM C266-89. Figure S4.a presents the setting times of RC-Cip, RC-BG-Cip, and RC-BG-Alg-Cip cements. The incorporation of BG into RC-Cip reduced both the initial and final setting times, confirming its accelerating effect on the setting reaction, in agreement with the XRD analysis of setting kinetics described in Section 2. In contrast, the addition of sodium alginate led to prolonged setting times, consistent with its role as a retardant. This effect is most likely associated with the ability of alginate to chelate free calcium ions as well as its viscosity, which restricts ionic diffusion pathways within the cement matrix.

The incorporation of ciprofloxacin also influenced the setting behavior. As shown in Figure S4.a, the addition of 3 wt% Cip significantly increased both the initial and final setting times across all cement formulations, highlighting the inhibitory effect of the drug on the setting reaction at low concentrations. This observation supports the inhibitory trends previously noted in the kinetics study.

Injectability results are shown in Figure S4.b. The incorporation of BG and alginate markedly improved injectability compared with RC-Cip, which is consistent with the improved cohesion behavior observed in the following tests. However, when Cip was added, injectability values decreased for all formulations, reflecting the drug’s adverse effect on paste fluidity.

Cohesion tests were performed by injecting fresh pastes (after 3 min of preparation) directly into PBS, and the samples were visually examined after 5 min and 24 h (Figure S4.c). RC-Cip displayed poor cohesion, fragmenting in PBS due to the filter-press phenomenon associated with its low injectability. The addition of BG improved cohesion, although slight disintegration was still observed (RC-BG-Cip). In contrast, the RC-BG-Alg-Cip composite showed excellent cohesion, with no visible turbidity or disintegration, and retained its integrity even after 24 h of immersion. This superior cohesion can be directly correlated with its high injectability (Figure S4.b).

The compressive strength of the cements was measured using an Instron 3369 Universal Testing Machine at a crosshead speed of 1 mm/min. Pastes were prepared at a liquid-to-powder ratio of 0.7 and cast into cylindrical silicone molds (8 mm × 16 mm). Results are shown in Figure S4.b. The incorporation of BG slightly reduced the compressive strength of RC-Cip from 2.9 MPa to 2.5 MPa. However, the RC-BG-Alg-Cip composite exhibited a significant enhancement, achieving a compressive strength of 10.3 MPa, approximately four times higher than that of RC and RC-BG formulations. This improvement can be attributed to the decrease in porosity and specific surface area, as confirmed by mercury porosimetry. The reinforcing effect of alginate appears to derive from its hydrogel-forming ability, which enhances paste cohesion and produces a denser microstructure, as observed in SEM images and consistent with porosity values (Figure 2 and Table 3 main manuscript).


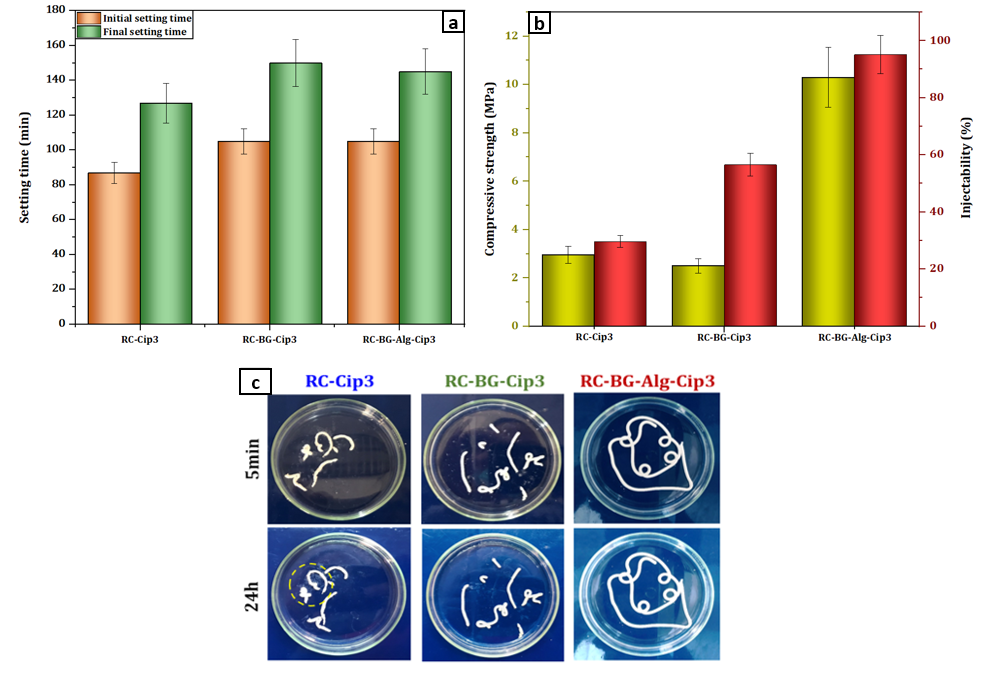


**Figure S4.** Effect of additives, BG and Alg, on the setting times (a), injectability, compressive strength (b), and cohesion (c).

**4- SEM-EDS analysis**

To further address the phase composition of the prepared cements, the Ca/P ratio was evaluated. Since ICP provides bulk elemental quantification, the resulting calcium and phosphorus contents would reflect the overall contribution of all phases present (precipitated apatite, residual vaterite, and bioactive glass), making the calculated ratio unsuitable for interpreting phase-specific stoichiometry. Instead, localized elemental analysis was carried out using EDS, which, although semi-quantitative, allows targeted point measurements within the cement matrix (Figure S5). The obtained Ca/P ratios (Table S1) were consistently higher than the theoretical value of stoichiometric apatite (1.67), indicating the coexistence of calcium-rich phases, such as residual vaterite and bioactive glass, alongside apatite. These results confirm that the global cement composition cannot be attributed to a single crystalline phase but rather to the combined contributions of the multiphase system.


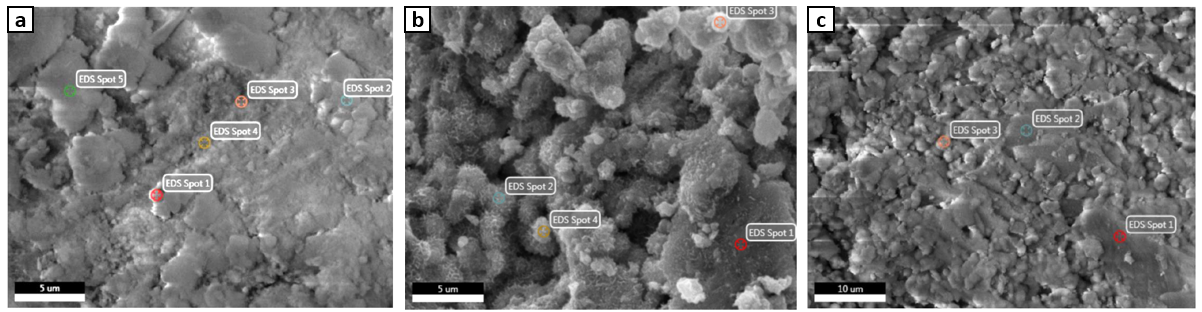


**Figure S5.** SEM-EDS analysis of RC, RC-BG, and RC-BG-Alg cements.

**Table S1.** Ca/P ratio determined from the EDS analysis for RC, RC-BG, and RC-BG-Alg cements.

|  | Ca/P | | |
| --- | --- | --- | --- |
| **EDS Spot** | **RC** | **RC-BG** | **RC-BG-Alg** |
| 1 | 2.547 | 1.997 | 2.959 |
| 2 | 2.554 | 2.233 | 2.661 |
| 3 | 2.624 | 2.582 | 3.700 |
| 4 | 2.617 | 2.233 |  |

**5- Bioactivity test**

Moreover, the bioactivity of the prepared cements was assessed by monitoring the formation of an apatite layer on their surface after immersion in SBF. Figure S6 presents representative SEM micrographs and FTIR spectra of the cement surfaces after 3 days of soaking. The SEM images reveal the deposition of agglomerated precipitates forming island-like structures, which are characteristic of apatite nucleation. This observation is consistent with the FTIR spectra, which exhibit bands assigned to phosphate and carbonate groups, confirming the precipitation of carbonated apatite on the cement surfaces.


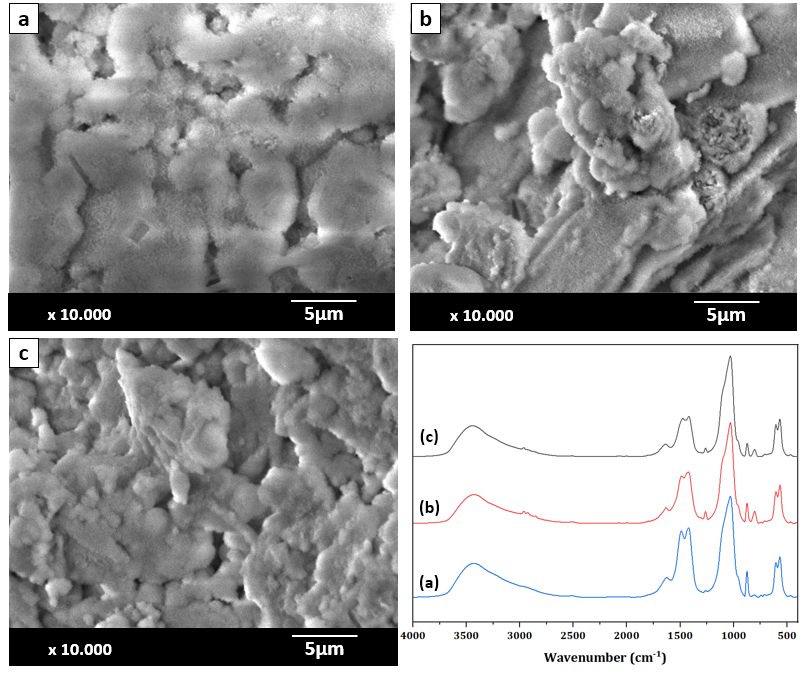


**Figure S6.** SEM micrographs of the cement surfaces after 3 days of immersion in SBF for RC-Cip (a), RC-BG-Cip (b), and RC-BG-Alg-Cip (c), along with their corresponding FTIR spectra (d).
